# Vinexin contributes to autophagic decline in brain ageing across species

**DOI:** 10.1038/s41418-021-00903-y

**Published:** 2021-11-30

**Authors:** So Jung Park, Rebecca A. Frake, Cansu Karabiyik, Sung Min Son, Farah H. Siddiqi, Carla F. Bento, Peter Sterk, Mariella Vicinanza, Mariana Pavel, David C. Rubinsztein

**Affiliations:** 1 Cambridge Institute for Medical Research, University of Cambridge, Cambridge, CB2 0XY UK; 2 UK Dementia Research Institute, Cambridge Biomedical Campus, Cambridge, UK; 3 Department of Immunology, Grigore T. Popa University of Medicine and Pharmacy of Iasi, Str. Universitatii 16, 700115 Iași, Romania

**Keywords:** Macroautophagy, Neural ageing

## Abstract

Autophagic decline is considered a hallmark of ageing. The activity of this intracytoplasmic degradation pathway decreases with age in many tissues and autophagy induction ameliorates ageing in many organisms, including mice. Autophagy is a critical protective pathway in neurons and ageing is the primary risk factor for common neurodegenerative diseases. Here, we describe that autophagosome biogenesis declines with age in mouse brains and that this correlates with increased expression of the *SORBS3* gene (encoding vinexin) in older mouse and human brain tissue. We characterise vinexin as a negative regulator of autophagy. *SORBS3* knockdown increases F-actin structures, which compete with YAP/TAZ for binding to their negative regulators, angiomotins, in the cytosol. This promotes YAP/TAZ translocation into the nucleus, thereby increasing YAP/TAZ transcriptional activity and autophagy. Our data therefore suggest brain autophagy decreases with age in mammals and that this is likely, in part, mediated by increasing levels of vinexin.

## Introduction

Macroautophagy (hereafter referred to as autophagy) is a highly conserved mechanism for maintaining cellular homoeostasis, which functions by trafficking cytoplasmic material for enzymatic degradation in the lysosome. In summary, phagophores engulf cytoplasmic cargo to form double-membraned autophagosomes, which fuse with lysosomes to form autolysosomes resulting in substrate degradation [1].

Increasing experimental evidence suggests autophagic decline is a ‘hallmark of ageing’, as it manifests during normal ageing, and amelioration extends healthy lifespan, while exacerbation accelerates age-related changes [2]. Autophagy was first linked to lifespan extension in the invertebrate *C. elegans*, with normal lifespan restored in long-lived daf-2 mutants when core autophagy genes are silenced [3]. More recently, lifespan extension has been observed in mice with increased autophagosome biogenesis driven either by overexpression of the core autophagy gene *Atg5* [4] or an activating knockin mutation in the autophagy regulator *Becn1* [5]. Mice with increased autophagy show amelioration of age-induced phenotypes, including improved glucose sensitivity and motor function [4], together with reduced fibrosis and apoptotic DNA fragmentation in the heart and kidney [5].

Reduced autophagosome formation and clearance is reported in liver tissue from mice aged to 20 months [6] and subsequently this finding has been replicated in aged mouse kidney and heart tissue [5]. However, neither reduced autophagosome biogenesis nor impaired flux through the autophagy pathway have previously been demonstrated in aged mammalian brain tissue. Autophagic decline is especially relevant to neurons, as post-mitotic cells are not able to segregate dysfunctional proteins and organelles from daughter cells using mitosis [7], which results in an increased reliance on autophagy to preserve cytoplasmic homeostasis. Consistent with this observation, dysfunctional autophagy is implicated in common, age-related neurodegenerative conditions such as Alzheimer’s disease and Parkinson’s disease [8]. Autophagy upregulation is therefore considered a potential therapeutic strategy in these conditions.

Here, we identify reduced autophagosome biogenesis in aged mouse brains that may be explained, at least in part, by increased expression of the multi-domain adaptor protein vinexin. We characterise vinexin (encoded by *SORBS3*), which modulates the actin cytoskeleton through various binding partners [9], as a negative regulator of autophagy and define the molecular mechanism. Vinexin depletion increases filamentous actin (F-actin) bundles, which compete with the transcriptional coactivators YAP and TAZ for binding to cytosolic angiomotins (AMOTs). This releases YAP/TAZ to enter the nucleus and increase transcriptional activity, thereby upregulating autophagy. We show *SORBS3* mRNA expression increases with age in mouse and human brain tissue. This corresponds to fewer autophagic vesicles in cerebral cortex samples from aged mice, as well as reduced mRNA expression of actin-related genes known to function in autophagosome biogenesis (*MLC2* and *MYH10*) [10] in older mouse and human brain tissue. Altogether, our data suggest increased *SORBS3* expression in ageing brains contributes to autophagic decline in mammalian brain ageing.

## Results

### Vinexin negatively regulates autophagy in multiple cell lines, including human neuroblastoma cells and mouse primary neurons

**Fig. 1:**
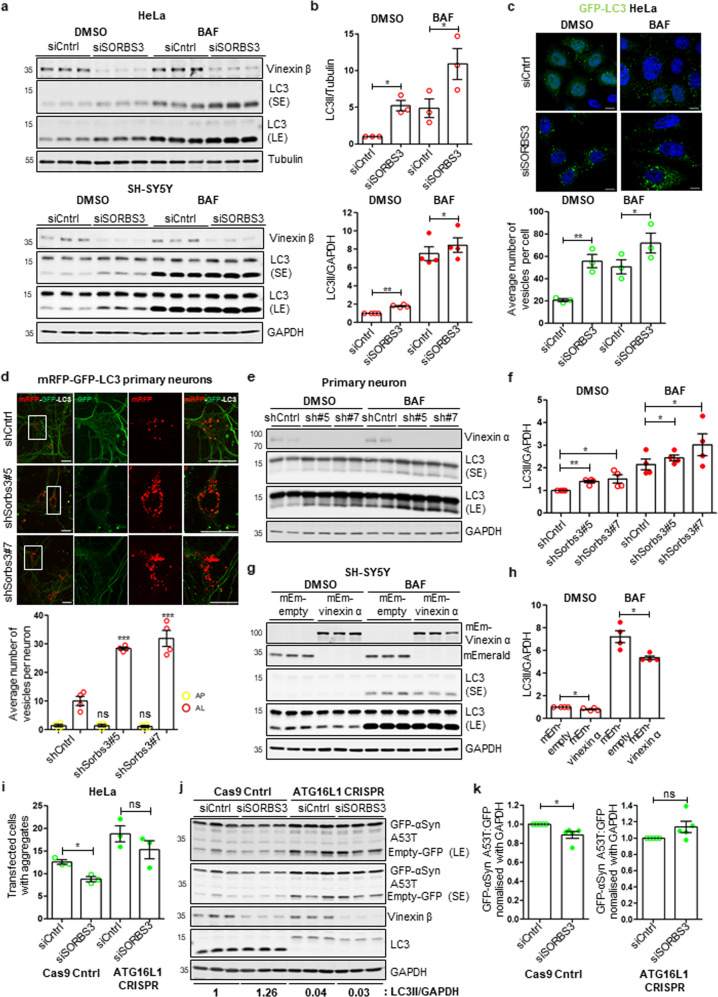
Vinexin negatively regulates autophagy. **a** HeLa and SH-SY5Y cells were depleted of vinexin beta using an individual siRNA oligonucleotide against SORBS3 (siSORBS3; oligo 7). Cells were incubated with bafilomycin A1 (BAF; 400 nM) or DMSO vehicle control for 4 h. Endogenous tubulin, GAPDH, LC3 and vinexin beta protein levels were examined by western blotting. Representative blots from three or four independent experiments per cell line are shown. SE = short exposure; LE = long exposure; molecular weights shown in kDa. **b** Quantification of 3 (HeLa) or 4 (SH-SY5Y) independent experiments per cell line. LC3-II (lower band of LC3 doublet) levels are expressed relative to loading control (tubulin or GAPDH) and normalised to LC3-II/tubulin or LC3-II/GAPDH in control siRNA (siCntrl) treated cells. * = *p* < 0.05; ** = *p* < 0.01 by two-tailed paired t-test. Error bars indicate SEM. **c** HeLa cells stably expressing GFP-LC3 were depleted of vinexin beta as in **a**. Cells were treated with BAF (400 nM) or DMSO vehicle control for 4 h. GFP-LC3 was examined by confocal microscopy. Representative images from 3 independent experiments are shown. Green = GFP; blue = DAPI. Scale bars indicate 10 µm. (bottom) GFP-LC3 puncta from the experiments described in **c**. were counted manually from confocal microscopy images. Quantification of three independent experiments is shown. * = *p* < 0.05; ** = *p* < 0.01 by two-tailed paired *t* test. Error bars indicate SEM. **d** (top) Mouse primary cortical neurons from mRFP-GFP-LC3 (tfLC3) transgenic mice were infected with either control shRNA (shCntrl) or shRNA against Sorbs3 (shRNA Sorbs3 #5 or #7) lentiviral vector. Scale bars indicate 20 µm. Representative images from four independent experiments are shown. (bottom) Manual quantification of GFP/mRFP-double positive vesicles (autophagosomes; AP) and mRFP-only vesicles (autolysosomes; AL) from the representative experiment shown in **d**. Autophagosomes and autolysosomes were first identified as mRFP positive vesicles. mRFP positive vesicles that overlapped with GFP positive vesicles were then counted as autophagosomes, while vesicles that were only positive for mRFP were counted as autolysosomes. In total, 25–35 cells analysed per condition per experiment in four independent experiments. ns = *p* > 0.05; *** = *p* < 0.001 by two-tailed Student’s *t* test. **e** Mouse primary cortical neurons from wild-type mice were infected with either control or Sorbs3 shRNA (sh#5 or sh#7) lentiviral vector. Infected cells were incubated with either DMSO or bafilomycin A1 (BAF; 400 nM) for 4 h. Endogenous LC3, GAPDH, and vinexin alpha protein levels were examined by western blotting. Representative blot from four independent experiments is shown. SE = short exposure; LE = long exposure; molecular weights shown in kDa. **f** Quantification of four independent experiments. LC3-II levels are expressed relative to GAPDH loading control and normalised to LC3-II/GAPDH in control shRNA (shCntrl) infected cells. * = *p* < 0.05; ** = *p* < 0.01 by two-tailed paired t-test. Error bars indicate SEM. **g** SH-SY5Y cells were transfected with either mEmerald-empty (mEm-empty) or mEmerlad-vinexin alpha (mEm-vinexin α). Transfected cells were treated with bafilomycin A1 (BAF; 400 nM) or DMSO vehicle control for 4 h. mEmerald, mEmerald-vinexin alpha, GAPDH and LC3 protein levels were examined by western blotting. Representative blots from 4 independent experiments per cell line are shown. SE = short exposure; LE = long exposure; molecular weights shown in kDa. **h** Quantification of four independent experiments. LC3-II levels are expressed relative to GAPDH loading control and normalised to LC3-II/GAPDH in control mEmerald-empty (mEm-empty) transfected cells. * = *p* < 0.05 by 2-tailed paired t-test. Error bars indicate SEM. **i** Autophagy-competent (Cas9 Cntrl) and autophagy-deficient (ATG16L1 CRISPR) HeLa cells were depleted of vinexin beta using an individual siRNA oligonucleotide against SORBS3 (siSORBS3; oligo 7). Cells were transfected with an aggregate-prone GFP-tagged huntingtin exon 1 fragment containing 74 glutamine repeats [GFP-Htt (Q74)] for 48 h. GFP-positive (total transfected) cells and cells with GFP-Htt aggregates were counted manually by fluorescence microscopy and the percentage of the total transfected cells with aggregates was calculated. *n* ≥ 600 transfected cells per condition. Quantification of three independent experiments is shown. ns = *p* > 0.05; * = *p* < 0.05 by two-tailed paired t-test. Error bars indicate SEM. **j** Cas9 Cntrl and ATG16L1 CRISPR HeLa cells were depleted of vinexin beta as in **a**, then transfected with both EGFP-tagged mutant alpha-synuclein (pEGFP-A53T α-syn) and pEGFP-empty vector (Empty-GFP). EGFP, EGFP-A53T α-syn, vinexin beta, GAPDH and LC3 protein levels were examined by western blotting. LC3-II/GAPDH protein levels are indicated below the corresponding lanes. Representative blot from five independent experiments per cell line is shown. SE = short exposure; LE = long exposure; molecular weights shown in kDa. **k** Quantification of 5 independent GFP-A53T α-syn degradation assays represented in **j**. The ratio of GFP-A53T α-syn to GFP level (nomalised to GAPDH) is shown for Cas9 Cntrl (left panel) and ATG16L1 CRISPR (right panel) HeLa cells. ns = *p* > 0.05; * = *p* < 0.05 by two-tailed paired t-test. Error bars indicate SEM.

During ongoing screening for autophagy modulators, we investigated vinexin using siRNA against *SORBS3* (si*SORBS3*) in HeLa (human cervical cancer), SH-SY5Y (human neuroblastoma) and RPE (human retinal pigment epithelium) cells. In all immortalised cell lines, vinexin beta (~37 kDa) was the only vinexin isoform expressed at the protein level and si*SORBS3* treatment caused a robust reduction in expression (Fig. 1a, Sup. Fig. 1a). We assayed autophagy by western blotting for the autophagic vesicle marker LC3-II in the presence and absence of bafilomycin A1 (BAF), which prevents autophagosome-lysosome fusion/LC3-II degradation [11]. si*SORBS3* increased LC3-II (lower band of LC3 doublet) under basal conditions (DMSO vehicle control) and in cells treated with BAF at a saturating concentration (Fig. 1a, b; Sup. Fig. 1a, b), which is consistent with increased autophagosome formation in vinexin beta-depleted cells [12]. In addition, increased GFP-LC3 positive vesicles (autophagosomes) were identified in vinexin beta-depleted HeLa cells stably expressing GFP-LC3 in both the presence and absence of BAF (Fig. 1c). We discriminated non-acidified autophagosomes from acidified autolysosomes using mRFP and GFP tandemly tagged to LC3 (mRFP-GFP-LC3), as the GFP signal is quenched by the acidic lysosomal pH relative to the mRFP signal [13]. si*SORBS3* increased both autophagosome (mRFP/GFP-double positive vesicle) and autolysosome (mRFP-single positive vesicle) numbers in HeLa cells stably expressing mRFP-GFP-LC3 (Sup. Fig. 1c, d). Similarly, autolysosome (mRFP-single positive vesicle) numbers and LC3-II levels were increased in mouse primary neurons treated with shRNA against *Sorbs3* (sh*Sorbs3*; Fig. 1d–f). This corresponded to a robust reduction in vinexin alpha (~75 kDa), which comprises vinexin beta plus an additional N-terminal SoHo domain [14], following treatment with sh*Sorbs3* oligos 5 and 7, the two oligos that effectively reduce vinexin alpha levels (Fig. 1e, f). Moreover, consistent with increased autophagosome biogenesis, the phagophore marker ATG16L1 [15] was increased in HeLa cells treated with si*SORBS3* (Sup. Fig. 1e, f).

We next addressed whether increased vinexin expression downregulated autophagy. Overexpressing mEmerald-tagged vinexin alpha decreased LC3-II in both DMSO and BAF-treated SH-SY5Y cells (Fig. 1g, h). LC3-II was also decreased in HeLa cells overexpressing mEmerald-vinexin alpha (Sup. Fig. 1g, h). We attempted to repeat these experiments with fluorescent- and epitope-tagged vinexin beta, but found the overexpressed vinexin beta consistently formed non-physiological protein aggregates. Since almost all vinexin binding partners utilise the three C-terminal SH3 domains common to both vinexin isoforms (as opposed to the relatively under characterised N-terminal SoHo domain unique to vinexin alpha) [9], we did not pursue experiments overexpressing vinexin beta further.

To allay concerns about possible siRNA off-target effects, HeLa cells were treated with two independent siRNA oligonucleotides targeting *SORBS3* (Sup. Fig. 2). Both increased endogenous LC3/CD63 colocalisation, indicating increased autolysosome formation (Sup. Fig. 2a–c). Moreover, mEmerald-vinexin alpha overexpression ameliorated the increase in endogenous LC3 puncta caused by si*SORBS3* (Sup. Fig. 2d, e).

Finally, we investigated whether vinexin beta depletion impacts clearance of disease-relevant autophagy substrates. The first such substrate we examined was the aggregate-prone model autophagy substrate GFP-Htt(Q74) (GFP-tagged huntingtin exon 1 fragment containing 74 glutamine repeats) [16]. si*SORBS3* treatment reduced the percentage of GFP-positive cells containing GFP-Htt(Q74) aggregates (which corresponds to autophagy substrate levels [16]) in autophagy-competent (Cas9 Cntrl) HeLa cells, but not autophagy-deficient HeLa cells lacking *ATG16L1* (*ATG16L1* CRISPR; Fig. 1i). We next expressed A53T mutant alpha-synuclein that causes Parkinson’s disease [17] and is also an autophagy substrate [18]. si*SORBS3* treatment reduced GFP-tagged alpha-synuclein A53T levels in Cas9 Cntrl HeLa cells, but not autophagy-deficient *ATG16L1* CRISPR HeLa cells (Fig. 1j, k). Taken together, these data suggest vinexin negatively regulates autophagy and impacts autophagic substrate levels in multiple experimental set-ups, including mouse primary cortical neurons

### Vinexin beta depletion upregulates autophagy through YAP/TAZ

**Fig. 2:**
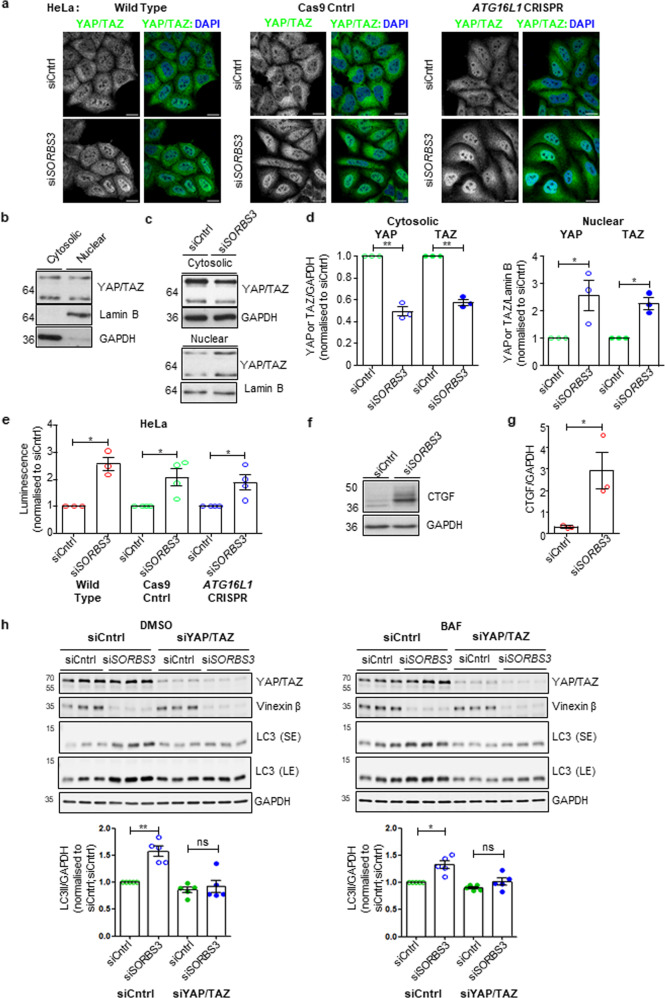
Vinexin beta depletion upregulates autophagy through YAP/TAZ. **a** Wild type, Cas9 control (Cntrl) and ATG16L1 CRISPR HeLa cells were depleted of vinexin beta using an individual siRNA oligonucleotide against SORBS3 (siSORBS3; oligo 7). Endogenous YAP/TAZ were examined by immunofluorescence and confocal microscopy. Representative images from three independent experiments per cell line are shown. Green = YAP/TAZ (Alexa Fluor 488); blue = DAPI. Scale bars indicate 20 µm. **b** Wild type HeLa cell lysate was subject to nuclear/cytosolic fractionation. Endogenous YAP/TAZ, lamin B and GAPDH protein levels in the nuclear and cytosolic fractions were examined by western blotting. Representative blot from the two independent experiments is shown. Molecular weights shown in kDa. **c** HeLa cells were depleted of vinexin beta using siSORBS3 (oligo 7) and lysates subject to nuclear/cytosolic fractionation and western blotting, as described in **b**. Representative blots from the two independent experiments in technical triplicate are shown. Molecular weights shown in kDa. **d** Quantification of the representative nuclear/cytosolic fractionation experiment in technical triplicate. YAP (upper band of YAP/TAZ doublet) and TAZ (lower band of YAP/TAZ doublet) are expressed relative to GAPDH (cytosolic fraction) or lamin B (nuclear fraction) loading control. YAP or TAZ/GAPDH or lamin B was normalised to control siRNA (siCntrl) treated cells. ns = *p* > 0.05, * = *p* < 0.05; ** = *p* < 0.01, ** = *p* < 0.001 by two-tailed paired t-test. Error bars indicate SEM. **e** Wild type, Cas9 control (Cntrl) and ATG16L1 CRISPR HeLa cells were depleted of vinexin beta, as described in **a**. Cells were co-transfected with synthetic TEAD (YAP/TAZ-responsive) promoter driving luciferase expression (pGL3b-8xGTIIC-luciferase) and Renilla luciferase control reporter for 24 h. Luminescence (firefly luciferase activity relative to Renilla luciferase activity) was measured by dual-luciferase reporter assay and normalised to control siRNA (siCntrl) treated cells. Quantification of 3 (wild type) or 4 (Cas9 Cntrl and ATG16L1 CRISPR) independent experiments is shown. * = *p* < 0.05 by two-tailed paired t-test. Error bars indicate SEM. **f** Wild type HeLa cells were depleted of vinexin beta, as described in **a**. Levels of CTGF protein (YAP/TAZ/TEAD direct target) and GAPDH (loading control) were examined by western blotting. Representative blot from the one experiment in biological triplicate is shown. Molecular weights are shown in kDa. **g** Quantification of 3 independent  experiments as shown in **f**. * = *p* < 0.05 by two-tailed Student’s t-test. Error bars indicated SD. **h** (top) HeLa cells were depleted of vinexin beta using an individual siRNA oligonucleotide against SORBS3 (siSORBS3; oligo 7) and YAP/TAZ using a pools of four siRNA oligonucleotides against YAP and TAZ (siYAP/TAZ). Cells were incubated with bafilomycin A1 (BAF; 400 nM) or DMSO vehicle control for 4 h. Endogenous YAP/TAZ, GAPDH, vinexin beta and LC3 and protein levels were examined by western blotting. Representative blots from five independent experiments are shown. SE = short exposure; LE = long exposure; molecular weights shown in kDa. (bottom) Quantification of five independent experiments described in **h**. LC3-II (lower band of LC3 doublet) levels are expressed relative to GAPDH loading control and normalised to LC3-II/GAPDH in siCntrl treated cells. ns = *p* > 0.05, * = *p* < 0.05; ** = *p* < 0.01 by two-tailed paired t-test. Error bars indicate SEM.

We and others have linked the transcriptional coactivators YAP and TAZ to both autophagosome formation [10] and autophagic flux [19, 20]. Moreover, YAP/TAZ transcriptional activity is influenced by actin cytoskeleton dynamics [21, 22], which are modulated by vinexin [9]. We therefore investigated whether altered vinexin expression impacted YAP/TAZ. Vinexin beta depletion using si*SORBS3* increased nuclear YAP/TAZ in HeLa cells (wild-type; Fig. 2a, Sup. Fig. 3), which is an accepted proxy measure of YAP/TAZ transcriptional activity [23]. In order to address the growing body of literature implicating autophagy (both directly and indirectly) in YAP/TAZ regulation [24–29], this result was confirmed in autophagy-deficient HeLa cells (*ATG16L1* CRISPR; Fig. 2a, Sup. Fig. 3). This finding suggests vinexin beta depletion promotes YAP/TAZ transcriptional activity independent of upregulating autophagy.

As expected, *SORBS3* knockdown also increased nuclear YAP/TAZ and decreased cytosolic YAP/TAZ levels in HeLa cells when examined biochemically using nuclear/cytosolic fractionation (Fig. 2b–d). This corresponded to increased YAP/TAZ transcriptional activity mediated through TEAD transcription factors, measured using a YAP/TAZ-responsive synthetic TEAD promoter driving luciferase expression. Luminescence was significantly higher in si*SORBS3* treated wild-type HeLa cells, together with *ATG16L1* CRISPR HeLa cells and autophagy-competent controls (Cas9 Cntrl; Fig. 2e). Expression of the YAP/TAZ-TEAD direct target gene *CTGF* also increased at the protein level in vinexin beta-depleted cells (Fig. 2f, g). These data support *SORBS3* knockdown promoting YAP/TAZ transcriptional activity independent of increasing autophagy.

To address whether vinexin regulates autophagy dependent on YAP/TAZ, HeLa cells were treated with si*SORBS3* in the presence and absence of siRNA against *YAP* and *TAZ*. *YAP/TAZ* knockdown ameliorated the increase in LC3-II observed in vinexin beta-depleted cells in both presence and absence of BAF (Fig. 2h). This finding suggests YAP/TAZ upregulation not only occurs upstream to autophagy in our system, but is necessary for increased autophagosome formation in vinexin beta-depleted cells.

The canonical autophagy signalling pathways (mTOR and ULK1) were not altered in si*SORBS3* treated cells (Sup. Fig. 4). Moreover, although vinexin is best known as a focal adhesion protein [9], we failed to find any causal relationship between focal adhesion changes in si*SORBS3* treated cells and autophagy (Sup. Figs. 5 and 6).

### Increased YAP/TAZ transcriptional activity and autophagosome formation upon *SORBS3* knockdown are F-actin dependent

**Fig. 3:**
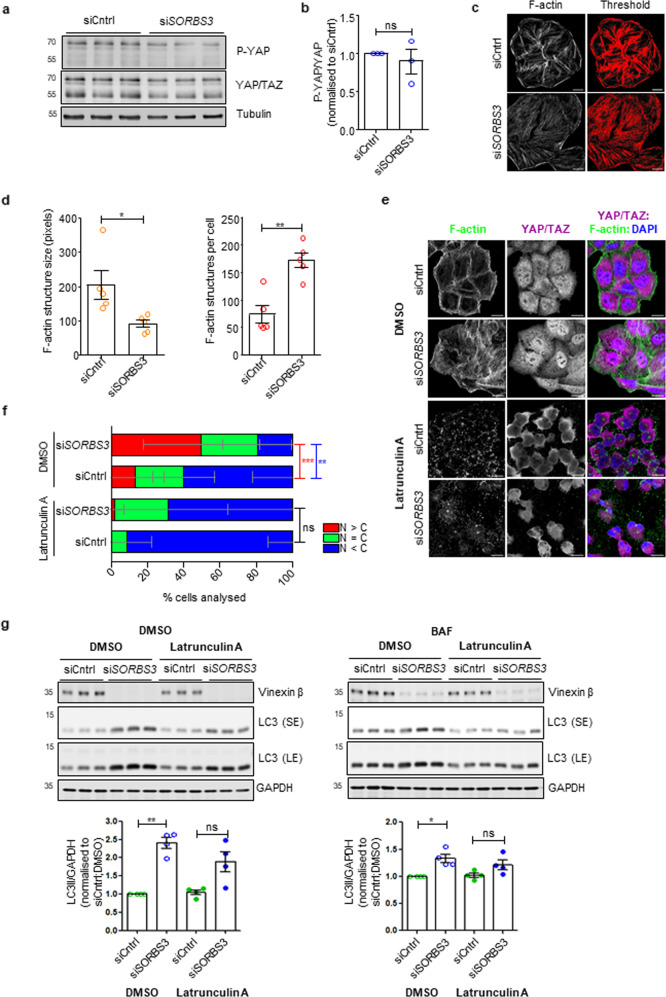
Vinexin depletion upregulates YAP/TAZ through a Hippo pathway-independent, filamentous actin-dependent mechanism. **a** HeLa cells were depleted of vinexin beta using an individual siRNA oligonucleotide against SORBS3 (siSORBS3; oligo 7). Endogenous total YAP/TAZ, YAP phosphorylated at serine 127, tubulin and vinexin beta protein levels were examined by western blotting. Representative blot from three independent experiments is shown. Molecular weights in kDa. **b** Quantification of three independent experiments. P-YAP and YAP (upper band of YAP/TAZ doublet) are expressed relative to tubulin loading control, the ratio of P-YAP/tubulin: YAP/tubulin taken and then normalised to P-YAP/YAP in siCntrl treated cells. ns = *p* > 0.05 by two-tailed paired t-test. Error bars indicate SEM. **c**. HeLa cells were depleted of vinexin beta as in **a**. Endogenous filamentous actin (F-actin) was visualised using Alexa Fluor 488-conjugated phalloidin and confocal microscopy. Confocal images (F-actin) were analysed using ImageJ software; F-actin structures were identified by thresholding (threshold). Representative images from five independent experiments are shown. Scale bars indicate 20 µm. **d** Quantification of F-actin structures per cell and F-actin structure size (pixels) from five independent experiments described in **c**. * = *p* < 0.05; ** = *p* < 0.01 by two-tailed paired t-test. Error bars indicate SEM. **e** HeLa cells were depleted of vinexin beta as in **a**. Cells were treated with latrunculin A (0.5 μM) or DMSO vehicle control for 6 h. Endogenous F-actin was visualised using Alexa Fluor 488-conjugated phalloidin and YAP/TAZ was examined by immunofluorescence. Representative confocal images from three independent experiments are shown. Green = F-actin (Alexa Fluor 488); purple = YAP/TAZ (Alexa Fluor 647); blue = DAPI. Scale bars indicate 20 µm. **f** Cells with predominantly nuclear YAP/TAZ (N > C), YAP/TAZ equally distributed between nucleus and cytosol (N = C) and predominantly cytosolic YAP/TAZ (C > N) where manually quantified. Quantification of the representative experiment shown in a. ns = *p* > 0.05; ** = *p* < 0.01; *** = *p* < 0.001 by two-tailed Student’s t-test. Red asterisks represent *p* value for N > C; blue asterisks represent N < C *p* value. *n* = 122 (siCntrl; DMSO); 75 (siSORBS3; DMSO); 165 (siCntrl; latrunculin A); 96 (siSORBS3; latrunculin A). Error bars indicate SD. **g** (top) HeLa cells were depleted of vinexin beta as in **a**. Cells were treated with latrunculin A (0.5 μM) or DMSO vehicle control for 2 h and then incubated with BAF (400 nM) or DMSO vehicle control for 4 h (total of 6 h treatment with latrunculin A or DMSO in the presence or absence of BAF). Endogenous GAPDH, LC3 and vinexin beta protein levels were examined by western blotting. Representative blots from four independent experiments are shown. SE = short exposure; LE = long exposure; molecular weights shown in kDa. (bottom) Quantification of four independent experiments described in **g**. LC3-II (lower band of LC3 doublet) levels are expressed relative to GAPDH loading control and normalised to LC3-II/GAPDH in DMSO treated siCntrl cells. * = *p* < 0.05; ** = *p* < 0.01 by two-tailed paired t-test. Error bars indicate SEM.

As the Hippo pathway is the principal YAP/TAZ regulatory mechanism [30], we examined Hippo pathway kinase activity in our system. To our surprise, si*SORBS3* treatment did not significantly alter YAP phosphorylation at the Hippo pathway kinase LATS1/2 target residue serine 127 (P-YAP; Fig. 3a, b).

Independent of the Hippo pathway, YAP/TAZ transcriptional activity increases with extracellular matrix stiffness, consequent to changes in actin cytoskeleton dynamics and independent of Hippo signalling [21, 22]. Using fluorophore-labelled phalloidin to visualise F-actin, we observed that vinexin beta depletion increased the numbers of F-actin structures per cell and decreased average F-actin structure size (Fig. 3c, d). The actin cytoskeleton modulator latrunculin A was used to address how this observation relates to increased YAP/TAZ transcriptional activity in cells treated with si*SORBS3*. Latrunculin A inhibits actin polymerisation through G-actin (monomeric actin) sequestration [31] and was used under conditions previously shown to downregulate YAP/TAZ (0.5 μM for 6 h) [22]. Latrunculin A treatment destroyed cytoskeletal F-actin in both vinexin beta-depleted cells and controls (Fig. 3e), which ameliorated the increase in YAP/TAZ nuclear localisation caused by si*SORBS3* (Fig. 3e, f). Similarly, latrunculin A treatment ameliorated the increase in LC3-II caused by vinexin beta depletion in HeLa cells in both the presence and absence of BAF (Fig. 3g). These data suggest that autophagy upregulation following si*SORBS3* treatment results from increased YAP/TAZ transcriptional activity, which is dependent on increased F-actin structures.

### Increased F-actin structures in vinexin beta-depleted cells upregulate autophagy by inhibiting YAP/TAZ cytosolic sequestration by angiomotins

**Fig. 4:**
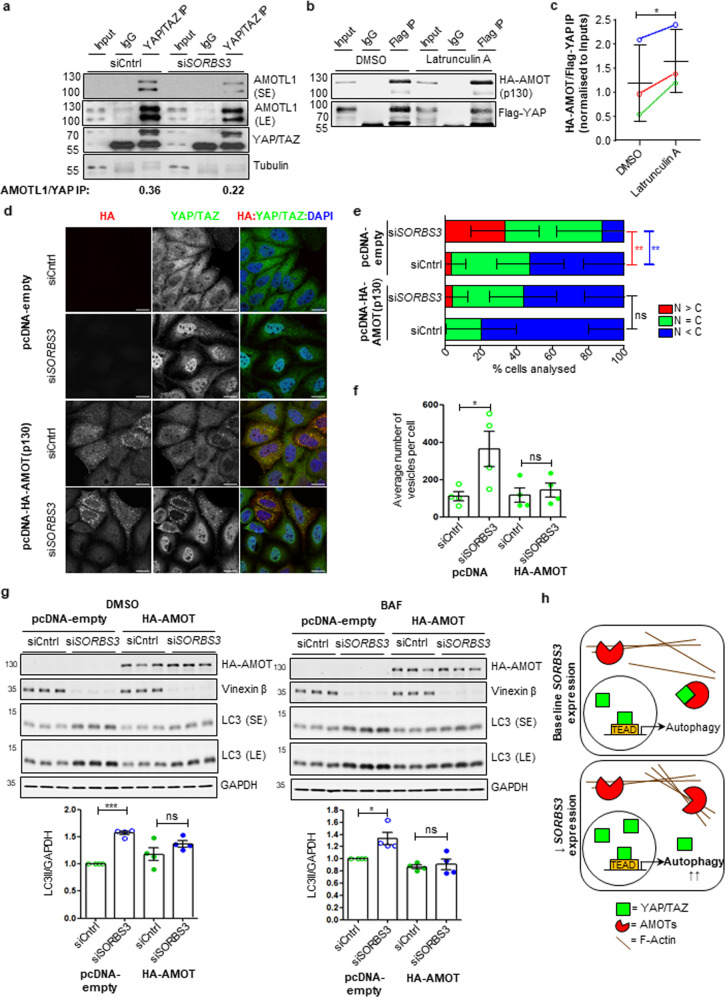
Vinexin depletion upregulates YAP/TAZ by countering YAP/TAZ cytosolic sequestration by angiomotins. **a** HeLa cells were depleted of vinexin beta using an individual siRNA oligonucleotide against SORBS3 (siSORBS3; oligo 7). Endogenous YAP/TAZ were immunoprecipitated using an antibody raised in mouse (YAP/TAZ IP). Normal mouse IgG was used as a negative control (IgG). Endogenous angiomotin-like protein 1 (AMOTL1), YAP/TAZ and tubulin were examined by western blotting. AMOTL1/YAP IP values are the ratio of AMOTL1 (both bands in doublet) to YAP in the in YAP/TAZ IP calculated after normalising AMOTL1 in YAP/TAZ IP to AMOTL1 in the input and YAP in the YAP/TAZ IP to YAP/TAZ in the input. TAZ is not quantified as obscured by IgG heavy chain in the IP. SE = short exposure; LE = long exposure; molecular weights shown in kDa. **b** HeLa cells were co-transfected with Flag-YAP and HA-AMOT(p130) for 48 h. Cells were treated with latrunculin A (0.5 μM) or DMSO vehicle control for 6 h. Exogenous Flag-YAP was immunoprecipitated using a mouse antibody against Flag (Flag IP). Normal mouse IgG was used as a negative control (IgG). Flag-YAP and HA-AMOT(p130) were examined by western blotting. Representative blot from three independent experiments is shown. Molecular weights shown in kDa. **c** Quantification of three independent experiments described in **b**. Amount of Flag-YAP and HA-AMOT(p130) in Flag IP are expressed relative to Flag-YAP and HA-AMOT(p130) in Input and HA-AMOT(p130) IP normalised to Flag-YAP IP. Independent experiment 1 is illustrated with green data points, experiment 2 with red data points and experiment 3 with blue data points. * = *p* < 0.05 by two-tailed paired t-test. Error bars indicate SEM. **d** HeLa cells were depleted of vinexin beta as in **a**. siCntrl and siSORBS3 treated cells were transfected with pcDNA-HA-AMOT(p130) or empty vector control (pcDNA-empty) for 24 h. Haemagglutinin (HA) and endogenous YAP/TAZ were examined by immunofluorescence. Representative confocal images from three independent experiments are shown. Red = HA (Alexa Fluor 568); green = YAP/TAZ (Alexa Fluor 488); blue = DAPI. Scale bars indicate 20 µm. **e** Cells with predominantly nuclear YAP/TAZ (N > C), YAP/TAZ equally distributed between nucleus and cytosol (N = C) and predominantly cytosolic YAP/TAZ (C > N) where manually quantified. Quantification of the representative experiment shown in **d**. ns = *p* > 0.05; ** = *p* < 0.01 by two-tailed Student’s t-test. Red asterisks represent *p* value for N > C; blue asterisks represent N < C *p* value. *n* = 56 (pcDNA-empty; siCntrl); 68 (pcDNA-empty; siSORBS3); 70 (pcDNA-HA-AMOT; siCntrl); 45 (pcDNA-HA-AMOT; siSORBS3). Error bars indicate SD. **f**. HeLa cells stably expressing GFP-LC3 were depleted of vinexin beta. siCntrl and siSORBS3 treated cells were transfected with pcDNA-HA-AMOT(p130) or empty vector control (pcDNA-empty) for 24 h. GFP-LC3 vesicles were counted manually. Quantification of 4 independent experiments is shown. ns = *p* > 0.05; * = *p* < 0.05 by two-tailed paired t-test. Error bars indicate SEM. **g** (top) HeLa cells were transfected with either siCntrl or siSORBS3 and then transfected with pcDNA-HA-AMOT(p130) or empty vector control (pcDNA-empty) for 24 h. Cells were treated with BAF (400 nM) or DMSO vehicle control for 4 h. Haemagglutinin (HA), GAPDH and LC3 protein levels were examined by western blotting. Representative blots from four independent experiments are shown. SE = short exposure; LE = long exposure; molecular weights shown in kDa. (Below). (bottom) Quantification of the four independent experiments is shown below. LC3-II (lower band of LC3 doublet) levels are expressed relative to GAPDH loading control and normalised to LC3-II/GAPDH in control siRNA (siCntrl) treated cells. ns = *p* > 0.05; * = *p* < 0.05; *** = *p* < 0.001 by two-tailed paired t-test. Error bars indicate SEM. **h** Schematic of proposed mechanism. In cells with reduced SORBS3 expression (↓ SORBS3 expression) relative to baseline (baseline SORBS3 expression) increased F-actin structures compete with YAP/TAZ for binding to angiomotins (AMOTs). Therefore, more YAP/TAZ is free to enter the nucleus and upregulate autophagy via increased YAP/TAZ/TEAD transcriptional activity.

The angiomotin family of proteins (AMOTs) binds YAP/TAZ in the cytosol, thereby inhibiting YAP/TAZ nuclear translocation, independent of Hippo signalling [32–34]. This interaction utilises AMOT L/PPxY motifs, which closely flank the AMOT F-actin binding region [35]. Thus, F-actin and YAP/TAZ compete for AMOT binding. We examined YAP/TAZ/AMOT binding by endogenous immunoprecipitation and found much less AMOTL1 (angiomotin-like protein 1) was pulled down with YAP/TAZ from HeLa cells following si*SORBS3* treatment (Fig. 4a). In order to confirm the role of F-actin, HeLa cells were co-transfected with Flag-tagged YAP (Flag-YAP) and haemagglutinin-tagged full-length AMOT p130 (HA-AMOT(p130)), then treated with latrunculin A or DMSO vehicle control. Significantly more HA-AMOT(p130) pulled down with Flag-YAP from latrunculin A treated cells (Fig. 4b, c). These data suggest increased F-actin structures in vinexin beta-depleted cells reduce YAP/TAZ/AMOT binding, thereby increasing YAP/TAZ nuclear translocation.

Using endogenous YAP/TAZ localisation as a proxy measure of YAP/TAZ transcriptional activity, we found that overexpression of AMOT p130 ameliorated the increased nuclear localisation of YAP/TAZ in si*SORBS3-*treated HeLa cells (Fig. 4d, e). This indicates that AMOT p130 overexpression saturates AMOT/F-actin binding (even in si*SORBS3-*treated cells with increased F-actin structures), leaving excess AMOTs free to retain YAP/TAZ in the cytosol.

AMOT p130 overexpression countered the increase in GFP-LC3 puncta resulting from si*SORBS3* treatment in HeLa cells stably expressing GFP-LC3 (Fig. 4f). Furthermore, AMOT p130 overexpression ameliorated the increase in LC3-II caused by vinexin beta depletion in HeLa cells in both the presence and absence of BAF (Fig. 4g). Taken together, these data support a molecular mechanism (summarised in Fig. 4h) in which vinexin beta depletion increases F-actin structures that compete with YAP/TAZ for AMOT binding in the cytosol. This facilitates YAP/TAZ transcriptional activity in the nucleus, leading to autophagy upregulation.

### Autophagy declines in aged mouse cerebral cortex, corresponding to increased *SORBS3* mRNA expression in mouse and human brain tissue

**Fig. 5:**
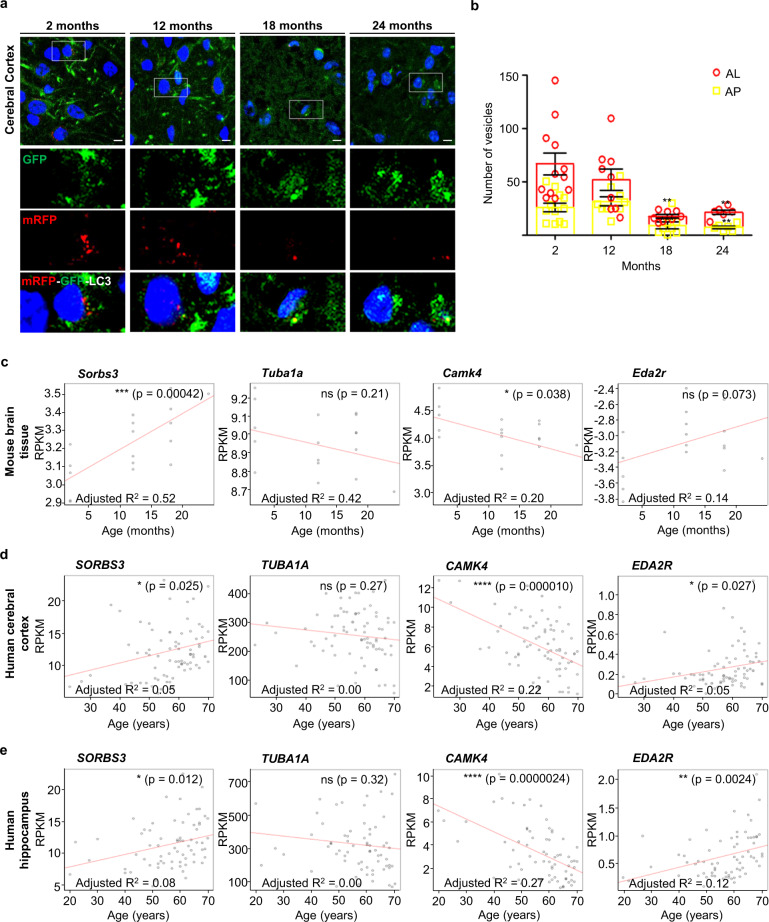
Autophagy declines with age in mouse motor cortex, corresponding to increased SORBS3 mRNA expression in mouse and human brain tissue. **a** Representative confocal images of sections of mouse motor cortex obtained from GFP-mRFP-LC3 mice aged to 2, 12, 18 and 24 months. **b** Manual quantification of autolysosomes (AL) and autophagosomes (AP) in the motor cortex of 2 (*n* = 12), 12 (*n* = 9), 18 (*n* = 8) and 20–24 (*n* = 3) months old GFP-mRFP-LC3 mice. Autophagic vesicles (autophagosomes and autolysosomes) were first identified as mRFP positive vesicles. mRFP positive vesicles that overlapped with GFP positive vesicles (GFP/mRFP-double positive vesicles) were counted as autophagosomes, while vesicles that were only positive for mRFP (mRFP-single positive vesicles) were counted as autolysosomes. ** = *p* < 0.01, *** = *p* < 0.001 by two-tailed Student’s t-test. Error bars indicate SEM. **c** Sorbs3 mRNA expression, Camk4 mRNA expression (positive control), Eda2r mRNA expression (positive control) and Tuba1a mRNA expression (negative control) determined by RNA sequencing of samples from wild-type mouse brain tissue (combined motor cerebral cortex, somatosensory cerebral cortex and striatum) plotted against chronological age of the animals in months. The adjusted coefficient of determination (adjusted R2) is displayed to two significant figures on the scatter plot (bottom left). Overall significance of the regression analysis was established by F-test and the *p* value displayed to two significant figures on the scatter plot (top right). * = *p* < 0.05, ** = *p* < 0.01, *** = *p* < 0.001, **** = *p* < 0.0001 by F-test. *n* = 18. RPKM = Reads Per Kilobase of transcript per Million. **d** SORBS3 mRNA expression, CAMK4 mRNA expression (positive control), EDA2R mRNA expression (positive control) and TUBA1A mRNA expression (negative control) determined by RNA sequencing of samples from ‘neuropathological normal’ human frontal cerebral cortex plotted against chronological age of the donors in years. Adjusted R2 is displayed to two significant figures (bottom left) and *p* value displayed to two significant figures (top right), as in **c**. *n* = 77. **e** SORBS3 mRNA expression, CAMK4 mRNA expression (positive control), EDA2R mRNA expression (positive control) and TUBA1A mRNA expression (negative control) determined by RNA sequencing of samples from ‘neuropathological normal’ human hippocampus were plotted against chronological age of the donors in years. Adjusted R2 is displayed to two significant figures (bottom left) and *p* value displayed to two significant figures (top right), as in **c**. *n* = 70.

Although impaired autophagy has previously been described in aged mouse liver, kidney and heart tissue [5, 6], to our knowledge autophagic vesicles and flux through the autophagy pathway have never been directly visualised in aged mammalian brain tissue. We therefore examined cortical sections from transgenic mice expressing mRFP-GFP-LC3 [36] aged to 2, 12, 18 and 24 months. Autophagic vesicles decreased with age in the mouse motor cortex, with significant reductions in autophagosomes (GFP/mRFP-double positive vesicles) and autolysosomes (mRFP-single positive vesicles) in mice aged to 18 months and a further significant reduction in autolysosomes in mice aged to 24 months (Fig. 5a, b). These data indicate autophagic decline in normal mouse brain ageing.

We explored whether this finding corresponded to increased *SORBS3* expression. Regression analysis was used to examine the relationship between chronological age and vinexin expression measured by RNA-Seq. *Sorbs3* mRNA expression in wild-type mouse brain tissue (combined motor cortex, somatosensory cortex and striatum) positively correlated with age (Fig. 5c). We successfully validated our approach using appropriate positive (*Camk4* and *Eda2r*) and negative (*Tuba1a*) controls (Fig. 5c) [37, 38]. The same approach was applied to RNA-Seq data from ‘neuropathologically normal’ human donors sourced through the GTEx consortium [39]. *SORBS3* mRNA expression positively correlated with age in human frontal cortex (Fig. 5d) and hippocampus (Fig. 5e), with the positive (*CAMK4* and *EDA2R*) and negative (*TUBA1A*) controls correlating as expected (Fig. 5d, e). These findings are consistent with previously published microarray analysis, which found increased *SORBS3* mRNA expression in older (≥70-year-old) human cerebral cortex, compared with younger (≤40-year old) cortex from an independent brain tissue set [38, 40].

**Fig. 6:**
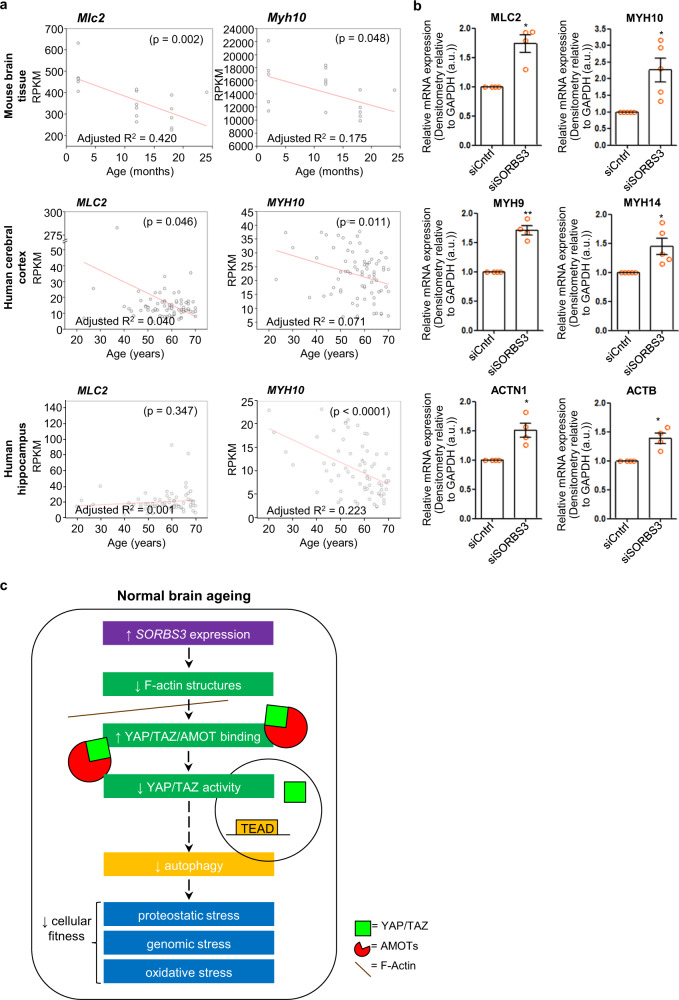
Expression of actin-related YAP/TAZ target genes negatively regulated by vinexin decreases with agein mouse and human brain tissue. **a** mRNA expression determined by RNA sequencing of actin-related YAP/TAZ target genes (*MLC2* and *MYH10*) in samples from wild-type mouse brain tissue (combined motor cerebral cortex, somatosensory cerebral cortex and striatum), ‘neuropathological normal’ human frontal cerebral cortex and ‘neuropathological normal’ human hippocampus plotted against chronological age in months (mice) or years (human). The adjusted coefficient of determination (adjusted R2) is displayed to two significant figures on the scatter plot (bottom left). Overall significance of the regression analysis was established by F-test and the *p* value displayed to two significant figures on the scatter plot (top right). *n* = 18 (mouse), 77 (human cerebral cortex), 70 (human hippocampus). **b** mRNA expression determined by RT-qPCR of actin-related genes (*MLC2, MYH10, MYH9, MYH14, ACTN1* and *ACTB*) in HeLa cells treated with either control siRNA (siCntrl) or SORBS3 siRNA (*siSORBS3*). Quantification of 4 (*MLC2, MYH9, ACTN1 and ACTB*) or 5 (*MYH10, MYH14*) independent experiments. * = *p* < 0.05, ** = *p* < 0.01 by two-tailed paired t-test. Error bars indicate SEM. **c** Schematic mechanism. Increased SORBS3 expression, as observed in normal brain ageing, decreases F-actin bundles. This enables angiomotins (AMOTs) to retain YAP/TAZ in the cytosol, which downregulates autophagy. Possible deleterious consequences, contributing to the age-related decline in ‘cellular fitness’ include impaired proteostasis, genomic instability stemming from crosstalk between autophagy and DNA repair mechanisms and reduced mitophagy causing oxidative stress.

Finally, we investigated how increased *SORBS3* mRNA expression impacted YAP/TAZ transcriptional activity in older mouse and human brain tissue. In keeping with our molecular mechanism (summarised in Fig. 6c), while *YAP1* and *TAZ* mRNA expression correlated positively with *SORBS3* expression (Sup. Fig. 7), expression of the known YAP/TAZ-TEAD target genes *MLC2*, *MYH10*, *BIRC2*, *ERBB4*, *RUNX2*, *CCND1* and *DAB2* [41–44] often correlated negatively with age in wild-type mouse brain tissue, human frontal cerebral cortex and human hippocampus, particularly *MYH10*, *BIRC2*, and *ERBB4* (Fig. 6a, Sup. Fig. 8). Notably, our lab has previously identified myosin light chain 2 (encoded by *MLC2*), myosin heavy chain 10 (encoded by *MYH10*) and several other actin-related genes *(MYH9, MYH14, ACTN1* and *ACTB)* as downstream targets of YAP/TAZ that function in autophagosome biogenesis [10].

To confirm the relationship between vinexin-regulated autophagy and actin-related YAP/TAZ target genes, we examined *MLC2*, *MYH10*, *MYH9*, *MYH14*, *ACTN1* and *ACTB* mRNA expression by RT-qPCR in si*SORBS3* treated HeLa cells. mRNA expression of these genes, known to function downstream to YAP/TAZ in autophagosome biogenesis [10], was significantly increased in HeLa cells treated with si*SORBS3* (Fig. 6b). Our data indicate *SORBS3* mRNA expression increases with age across human brain regions (frontal cortex and hippocampus) and across species (human and mouse). Given this occurs alongside reduced autophagy (measured in mouse motor cortex), as well as reduced YAP/TAZ-TEAD target gene expression in mouse and human brain tissue, vinexin is a likely contributor to autophagic decline in normal brain ageing via decreased YAP/TAZ transcriptional activity.

## Discussion

Vinexin was previously identified as a candidate autophagy regulator from a genome-wide image-based siRNA screen that suggested si*SORBS3* treatment increased autophagosome numbers due to decreased lysosomal processing, implying compromised autophagic flux [45]. While the previous data were not followed-up, our data in multiple cell lines, including mouse primary neurons, indicate that vinexin depletion using multiple reagents increases autophagic flux and substrate clearance by promoting autophagosome biogenesis. We have defined a pathway (summarised in Fig. 4h) whereby *SORBS3* knockdown increases F-actin structures, which compete with YAP/TAZ for binding to AMOTs in the cytosol. This promotes YAP/TAZ translocation into the nucleus, increasing YAP/TAZ transcriptional activity and thence autophagy.

We identify fewer autophagosomes and autolysosomes in motor cortex from mice aged to 18 and 24 months. This sits alongside increased *SORBS3* mRNA and YAP/TAZ target gene mRNA expression in brain tissue (combined motor cortex, somatosensory cortex and striatum) from aged mice, as well as frontal cortex and hippocampus from older human donors. We therefore hypothesise that increased vinexin expression contributes to autophagic decline via decreased YAP/TAZ transcriptional activity in normal brain ageing (Fig. 6c). The mechanism driving increased *SORBS3* mRNA expression in older mammalian brains remains to be explored. Moreover, vinexin is certainly not the only contributor to brain autophagy decreasing with age, as lower core autophagy gene (*ATG5*, *ATG7* and *BECN1*) expression has been observed in cerebral cortex from older human donors [40, 46].

Impaired autophagy in normal brain ageing is thought to contribute to an age-related decline in ‘cellular fitness’. Mechanisms attributable, at least in part, to reduced autophagy include impaired proteostasis causing toxic protein species to accumulate in the cytoplasm, genomic instability stemming from crosstalk between autophagy and DNA repair mechanisms and reduced abnormal mitochondria clearance by autophagy (mitophagy) leading to increased oxidative stress [7, 47, 48]. In all cases, neurons (as post-mitotic cells) are especially vulnerable, being unable to divide to relieve proteostatic, genomic or oxidative stress [7]. Impaired autophagy is implicated in common neurodegenerative conditions such as Alzheimer’s disease and Parkinson’s disease [8], while ageing is the primary risk factor for these conditions.

Our findings are also interesting to consider from an evolutionary standpoint. If increased vinexin expression does contribute to a deleterious reduction in autophagy in ageing brain tissue, why does this persist across species (mouse and human) and over evolutionary time? The simplest explanation is that the strength of natural selection declines dramatically with respect to genes influencing outcomes subsequent to an organism’s reproductive years. Another intriguing possibility is that *SORBS3* exhibits antagonistic pleiotropy and causes a ‘fitness trade-off’ [49]. Specifically, *SORBS3* has been identified as a tumour suppressor in hepatocellular carcinoma [50, 51], while our findings indicate increased *SORBS3* expression could promote an age-related decline in cellular (and especially neuronal) fitness.

## Materials and methods

### Antibodies

The following primary antibodies were used for western blot and immunofluorescence: rabbit polyclonal vinexin (alpha and beta isoforms) (ab126971, WB 1:500), rabbit monoclonal AMOTL1 (ab171977, WB 1:1000), mouse monoclonal GAPDH (ab8245, WB 1:3000), rabbit polyclonal Lamin B (ab16048, 1:1000), mouse monoclonal CD63 (ab8219, IF 1:100) from Abcam, Goat polyclonal CTGF (sc-14939, WB 1:200), mouse monoclonal YAP/TAZ (sc-101199, WB 1:200, IF 1:50) from Santa Cruz. Rabbit polyclonal ERK1/2 (9102, WB 1:1000), rabbit polyclonal p70S6K (9202, WB 1:1,000) rabbit polyclonal P-p70S6K (T389) (9205, WB 1:1,000), rabbit polyclonal ULK1 (4773, WB 1:1000), rabbit polyclonal P-ULK1 (S556) (5869, WB 1:1,000), rabbit polyclonal P-ULK1 (S758) (14202, WB 1:1000), rabbit monoclonal P-YAP (S127) (13008, WB 1:1000), rabbit monoclonal ATG16L1 (8089, IF 1:100) from Cell signalling. Rabbit polyclonal actin (A2066, Sigma-Aldrich, WB 1:2000), mouse monoclonal Flag (A2220, Sigma-Aldrich, 1:1000), rabbit polyclonal HA (H6908, Sigma-Aldrich, WB 1:1000, IF 1:100), mouse monoclonal tubulin (T9026, Sigma-Aldrich, 1:10000), rabbit polyclonal GFP (632592, Clontech, 1:10000), rabbit polyclonal LC3 (NB100-2220, Novus Biologicals, WB 1:10000), mouse monoclonal paxillin (610619, BD Biosciences, WB 1:1000, IF 1:100), mouse monoclonal vinculin (MAB3574, Millipore, IF 1:100).

The secondary antibodies for immunofluorescence were conjugated to Alexa Fluor 488, 568, 594 or 647 (Invitrogen). For western blotting, the following horseradish peroxidase (HRP)-conjugated secondary antibodies were used: anti-mouse (NA931V, GE Healthcare), anti-rabbit (NA934V, GE Healthcare) and anti-goat (611620, Invitrogen-Life Technologies). Alternatively, the following LICOR secondary antibodies were used: anti-mouse 680 and anti-rabbit 800. F-actin was visualised with Phalloidin-Alexa Fluor 546 (A22283, Invitrogen-Life Technologies) and 488 (A12379, Invitrogen-Life Technologies).

### Plasmids and siRNAs

The following pre-designed siRNAs (On-Target Plus SMART pool and/or set of deconvoluted oligos) were used: control (siCntrl, D-001810-10), SORBS3 SMARTpool (si*SORBS3* pool, L-015415-00), si*SORBS3* oligo 5 (J-015415-05) and oligo7 (J-015415-07), paxillin SMARTpool (si*PXN*; L-005163-00), YAP SMARTpool (si*YAP*; L-012200-00) and TAZ SMARTpool (si*TAZ*; L-016083-00). All siRNA was purchased from Dharmacon (GE Healthcare). siRNA was resuspended in siRNA buffer (Dharmacon B-002000-UB) to a final concentration of 20 μM, as per the manufacturer’s instructions.

The following constructs were used in this study: the EGFP-tagged huntingtin exon 1 fragment containing 74 glutamine repeats (EGFP-Htt(Q74)) was produced by our group [52], as was the EGFP-tagged mutant alpha-synuclein (pEGFP-A53T α-syn) [53]. EGFP-empty (EGFP-C1) was purchased from ClonTech. pcDNAempty (pcDNA3.1) was purchased from ThermoFisher (V79020). mEmerald-empty (mEmerald-C1; 53975) and mEmerald-vinexin alpha (mEmerald-Vinexin-C-14; 54305)[54], Flag-YAP (p2xFlag CMV2YAP2; 19045) [55], pcDNA-HA-AMOT(p130) (HA-AMOT p130; 32821) [34], pGL3b-8xGTIIC luciferase (8xGTIIC-luciferase; 34615) [22] were obtained from Addgene. The Renilla luciferase control reporter (pRL-CMV) was purchased from Promega (E2261).

Pre-designed pLKO.1 shRNAs vectors were acquired from The RNAi Consortium (TRC) (empty vector control; #RHS4080, Dharmacon; mouse *Sorbs3* shRNAs (sh#5; TRCN0000112956, sh#6: TRCN0000112957, sh#7; TRCN0000112958, sh#8; TRCN0000112959). Although all the shRNAs provided by TRC were validated, two single shRNAs were selected and used to perform the autophagic flux experiments in tfLC3 neurons (Sorbs3 sh#5; TRCN0000112956, sh#7; TRCN0000112958).

### Reagents

Bafilomycin A1 (BAF; Sigma-Aldrich 19-148) was resuspended in DMSO from Sigma-Aldrich and used at 400 nM for 4 h to block flux through the autophagy pathway. Latrunculin A (Santa Cruz, sc-202691) was resuspended in DMSO. In order to inhibit actin polymerisation, cells were treated with latrunculin A at 0.5 µM (diluted in complete media) for 6 h. In experiments using BAF and latrunculin A an equivalent volume of DMSO was used as the vehicle control.

### Cell culture

HeLa (human cervical adenocarcinoma) cells (validated by STR profiling and purchased from ATCC [American Type Culture Collection]), SH-SY5Y (human neuroblastoma; ATCC), RPE (human retinal pigment epithelium; ATCC) and HEK293T (human embryonic kidney 293T; ATCC) cells were maintained in high glucose (4500 mg/L) DMEM (Dulbecco’s Modified Eagle Medium; Sigma-Aldrich D6546) completed with 10% foetal bovine serum (Sigma-Aldrich F7524), 100 units/mL penicillin-streptomycin (Sigma-Aldrich P0781) and 2 mM L-glutamine (Sigma-Aldrich G7513) at 37 °C, 5% carbon dioxide. HeLa cells stably expressing GFP-LC3 and GFP-mRFP-LC3 have been described previously [13]. These cells were cultured in high glucose, complete DMEM (as described above) supplemented with 500 μg/mL G418 (Gibco 1181-031). Autophagy-deficient ATG16L1 CRISPR HeLa cells, together with autophagy-competent controls (Cas9 Cntrl HeLa), were generated by our group following previously published protocols [56, 57]. These cells were maintained in high glucose, complete DMEM (as described above). All cell lines were incubated at 37 °C and 5% CO_2_, humidified atmosphere and were regularly tested for mycoplasma contamination every two weeks.

### mRFP-GFP-LC3 (tfLC3) transgenic mice

The mRFP-GFP-LC3 reporter mouse line was generated in our lab as previously described [36]. All studies and procedures were performed with the jurisdiction of appropriate UK Home Office Project and Personal animal licences and with the approval of the University of Cambridge Animal Welfare and Ethical Review Body for animal study.

### Isolation and culture of primary mouse cortical neurons

For primary mRFP-GFP-LC3 (tfLC3) cortical neurons, transgenic mice were crossed with C57BL/6 mice (Jackson laboratories) and females which are at E16.5 gestation were sacrificed and embryos were harvested. Isolated cortices from all embryos (regardless of genetic status) were combined to create mixed cultures). Briefly, brains were harvested and placed in Hank’s Balanced Salt Solution (HBSS) where the meninges were removed and the cerebral cortices were dissected. After incubation in HBSS with 0.25% trypsin (Gibco) for 20 min at 37 °C, dissociated neurons were resuspended in HBSS and seeded on poly-D-lysine-coated MatTek glass-bottom culture dish (P35G-1.0-14-C) or 12 well plates. Cells were maintained in Neurobasal-A medium (#12349015, Thermo Fisher Scientific) supplemented with 2 mM GlutaMAX, 200 mM B27 supplement and 1% Penicillin–Streptomycin) at 37 °C and 5% CO_2_ in a humidified incubator. One-half of the culture medium was replenished with pre-warmed fresh medium every 2 days until infection. After 5 days of in vitro culturing, the differentiated neurons were infected with lentiviral particles for knockdown experiments.

### siRNA and DNA transfection

For siRNA transfection, HeLa and RPE cells were seeded in 6 well plates and cells were transfected with 80 nM of siRNA using Lipofectamine 2000 (ThermoFisher 11668019) and cells were post-transfected with 80 nM of siRNA using Lipofectamine 2000. SH-SY5Y cells were transfected with 80 nM of siRNA using Lipofectamine RNAiMAX (13778150 Invitrogen) for 4 h per well in six well plates. After a day, cells were post-transfected with 80 nM of siRNA using Lipofectamine RNAiMAX. After transfections, cells were incubated with full growth medium for 2 days. Transfected cells were split into 6 or 12 well plates as per experimental requirements.

For DNA transfection, HeLa and SH-SY5Y cells were plated in six well plates and cells were transfected with 1 μg DNA using TransIT 2020 reagent (Mirus MIR5400) according to the manufacturer’s instructions. SH-SY5Y cells were post-transfected with 1 μg DNA using TransIT 2020 reagent after 24 h. On the following day, cells were re-seeded in the 6 or 12 well plates as per experimental requirements.

### shRNA lentivirus production and infection

shRNA lentiviral particles were produced and transduced following the RNAi Consortium (TRC) protocols. Briefly, HEK293T packaging cells in 100 mm dishes were transfected at 50–60% of confluence with a mix of 2.5 μg psPAX2 vector (packaging vector), 270 ng pMD2.G vector (envelope vector) and 2.7 µg hairpin-pLKO.1 vector using TransIT-LT1 (Mirus) transfection reagent according to the manufacturer’s instructions. Transfected cells were cultured in high-serum medium. After 40 h, cell culture medium was harvested and replaced by high-serum medium at three times repeatedly for 24 h intervals. Viral preps were then concentrated by centrifugation at 160,100 *g* for 90 min.

For infection in primary neurons, viral titres were added to the cells in the presence of 6 μg/ml polybrene (Sigma Aldrich) and incubated overnight. On a following day, medium was replaced by full medium and cells were further incubated for an additional 3-4 days before testing the knockdown effects.

### Immunofluorescence microscopy

Cells were cultured on glass coverslips for experimental requirements. For immunostaining of ATG16L1, HA, paxillin and YAP/TAZ, cells were fixed with PFA (paraformaldehyde) at 37 °C: 2 min in 2% PFA (4% PFA in PBS mixed 1:1 with complete media), then 5 min in 4% PFA in PBS. For immunostaining of LC3 and CD63, cells were fixed with cold methanol for 5 min. In both cases, fixation was followed by permeabilisation using 0.1% Triton X-100 in PBS for 5 min at room temperature. Coverslips were then incubated in BSA (bovine serum albumin) blocking buffer (0.5% BSA in PBS, 50 mmol ammonium chloride) for at least 30 min at room temperature. For vinculin immunostaining, coverslips were first washed in Buffer C (100 mM PIPES pH 6.9, 0.5 mM MgCl_2_, 0.1 mM EDTA, 0.01 M EGTA), then fixed with Buffer D for 15 min at room temperature (1.4% PFA, 0.2% Triton X-100, 2 M Glycerol in Buffer C). Blocking was performed with 0.5% BSA in Buffer C (supplemented with 50 mmol ammonium chloride) for at least 30 min at room temperature. In all experiments, coverslips were incubated with primary antibody diluted in the appropriate blocking buffer for 4 h at room temperature or 18 h at 4 °C. Coverslips were then incubated with Alexa Flour-conjugated secondary antibodies (purchased from Thermo Fisher) in the appropriate blocking buffer for 1 h at room temperature protected from light. In order to detect F-actin, Alexa Flour 488 phalloidin (Thermo Fisher A12379) was added at the blocking step (diluted 1:500 in the appropriate blocking buffer). Coverslips were mounted using ProLong Gold Antifade with DAPI (Thermo Fisher P36935) and slides stored in the dark at 4 °C.

Imaging was performed using an LSM710, LSM780 and LSM880 confocal microscopes (40×, 63× NA 1.4 Plan Apochromat oil immersion lens, Carl Zeiss) in conjunction with ZEN software (black edition; Carl Zeiss).

### Imaging of mRFP-GFP-LC3 (tfLC3) primary neurons

For live-cell imaging of mRFP-GFP-LC3 primary neurons, primary neurons were monitored at DIV9-11 using LSM780 confocal microscopy (63× NA 1.4 Plan Apochromat oil-immersion lens; Carl Zeiss). At least ten fields were imaged and analysed using ZEN software. Autophagic vesicles (autophagosomes and autolysosomes) were first identified as mRFP positive puncta. Subsequently, mRFP positive puncta that overlapped with GFP positive puncta (GFP/mRFP-double positive vesicles) were counted as autophagosomes, while puncta that were only positive for mRFP (mRFP-single positive vesicles) were counted as autolysosomes.

### Cell lysis and western blot analysis

Cells were plated in 6 or 12 well plates according to experiment requirements. Cells were washed in cold phosphate-buffered saline (PBS) twice, then lysed directly in 1× Laemmli buffer (62.5 mM Tris pH 6.8, 2% w/v SDS, 10% glycerol, 50 mM DTT, 0.01% w/v bromophenol blue), supplemented with protease inhibitor cocktail (Sigma Aldrich 11873580001). Samples were boiled for 10 min at 100 °C.

For western blot analysis, samples were separated by SDS-PAGE gel electrophoresis and proteins were transferred to PVDF membranes (Millipore IPFL00005 or IPVH00005). The membranes were blocked with 5% non-fat milk in PBS/0.01% Tween for 1 h at room temperature. Membranes were subsequently incubated with primary antibodies for at least 12 h at 4 °C or 4 h at room temperature, then secondary antibodies for 1 h at room temperature. All antibodies were diluted in the appropriate blocking buffer. Proteins on the membrane were visualised either directly on an Odyssey Imager (LI-COR) or with electrochemiluminescent detection reagents (GE Healthcare RPN2232). Protein quantification was performed using Image Studio Lite software.

### Immunoprecipitation

For immunoprecipitation, cells were cultured in 25 cm^2^ dishes and lysed on ice in 1 mL buffer (50 mM Tris pH 7.5, 150 mM NaCl, 1 mM EDTA, 0.5% Triton X-100) supplemented with protease and phosphatase (Sigma Aldrich P5726 and P0044) inhibitor cocktails. Lysates were centrifuged at 15,000 rpm for 15 min at 4 °C. The pellet was discarded and protein concentration of the supernatant determined by Bradford protein assay (Bio-Rad 500-0201). Samples containing 1 mg total protein were incubated with primary antibodies diluted 1:200 for at least 18 h on a rotator mixer at 4 °C: mouse anti-YAP/TAZ (Santa Cruz sc-101199), mouse anti-Flag (Sigma-Aldrich A2220) and rabbit anti-HA (Sigma-Aldrich H6908). Samples from each experimental condition were also incubated with normal mouse or rabbit IgG (also diluted 1:200; Santa Cruz sc-2025 or sc-2027) to control for non-specific binding. Precipitated immunocomplexes were separated by incubation with Protein G magnetic beads (Dynabeads Protein G; Thermo Fisher 10004D) for 4 h on a rotator mixer at 4 °C. Input samples were stored at 4 °C during this time. All samples (immunoprecipitant and input) were boiled in 1× Laemmli buffer for 4 min at 100 °C, then examined by western blotting.

### GFP-Htt (Q74) aggregation assay

Cells cultured on glass coverslips in six-well plates were transfected with 1.5 µg GFP-Htt(Q74). After 48 h, coverslips were fixed with 4% PFA in PBS for 10 min at room temperature and mounted using ProLong Gold Antifade with DAPI. Slides were labelled so as to conceal coverslip identity. Percentage transfected (GFP-positive) cells containing aggregates were scored using an Eclipse E600 fluorescence microscope (Nikon). At least 200 cells were analysed per coverslip, with coverslips prepared in triplicate for each experimental condition.

### Alpla-synuclein A53T assay

Cells were transfected with both 1.5 µg of pEGFP-α-synuclein A53T and 0.5 µg of empty pEGFP per a well of a six-well plate. After 2 days, transfected cells were lysed and EGFP-α-synuclein A53T and GFP levels examined by western blotting (as described above). The ratio between both signals was calculated, normalised to GAPDH levels.

### Cytoplasmic/nuclear fractionation

Subcellular fractionation was performed as previously described [58]. In summary, cells were lysed with Buffer A (10 mM HEPES, 10 mM KCl, 0.1 mM EDTA, 0.4% NP-40, 1 mM DTT) supplemented with protease and phosphatase inhibitor cocktails on ice for 30 min. Lysates were centrifuged at 15,000 rpm for 10 min at 4 °C. Supernatants containing cytosolic proteins were collected and stored at 4 °C. Nuclear pellets were resuspended in buffer B (20 mM HEPES, 0.4 M NaCl, 1 mM EDTA, 10% glycerol, 1 mM DTT) supplemented with protease inhibitor cocktail and incubated on ice for 1 h. After centrifugation at 15,000 rpm for 10 min at 4 °C, supernatants containing nuclear proteins were collected. Protein concentrations were determined by Bradford protein assay. Both fractions were analysed by western blotting using primary antibodies against YAP/TAZ, Lamin B and GAPDH. Lamin B was used as a nuclear control and GAPDH as a cytosolic control.

### Luciferase reporter assay

Luciferase reporter assay was examined by Dual-Glo luciferase assay kit (Promega E1910). Cells cultured in six-well plates were transfected with 0.4 µg pGL3b-8xGTIIC-luciferase and 40 ng pRL-CMV (renilla luciferase). After 24 h post-transfection, cells were lysed in 300 µL ‘Passive Lysis Buffer’ per well for 20 min at room temperature. Lysates were centrifuged at 15,000 rpm for 5 min at 4 °C. The pellets were discarded and 10 µL of each supernatant combined with 50 µl ‘Luciferase Assay Buffer II’ in white 96-well plates. Firefly luminescence was measured using a GloMax 96 Microplate Luminometer (Promega). 50 µl ‘Stop & Glo Buffer’, prepared following the manufacturer’s instructions, was subsequently added to all wells. After 5 min, Renilla luminescence was measured using the GloMax 96 Microplate Luminometer. Firefly luciferase activity relative to Renilla luciferase activity was calculated as a ratio for each well.

### RNA isolation and quantitative real-time PCR

Total RNA was isolated from HeLa cells treated with either si*Cntrl* or si*SORBS3* using RNeasy Mini Kit (74104 Qiagen). The extracted RNA was used for cDNA synthesis using SuperScript III First Strand Synthesis System for RT-PCR (1880-051 Invitrogen) according to manufacturer’s instructions. The cDNA was mixed with PowerUp SYBR Green Master Mix (A25742 Applied Biosystems), forward primer (Fw) and reverse primer (Rv) for each target gene sequences (*MLC2*, *MYH10*, *MYH9*, *MYH14*, *ACTN1* and *ACTB*) purchased from Invitrogen. Real-time qPCR was then performed using Bio-Rad CFX96 Real-Time PCR detection system (1845096 Rio-Rad) following the manufacturer’s instructions.

The following primer pairs were used: Human *MLC2*, Fw: 5′-TTTGGGGAGAAGCTGAACGG-3′ Rv: 5′-TCATGGATGAAACCTGAGGC-3′; Human *MYH9*, Fw: 5′-GGAAGGCTAAGCAAGGCTGA-3’ Rv: 5′-ACTTATAGCCAGGACCTGAACC-3′; Human *MYH10*, Fw: 5′-GACTGAGGCGCTGGATCTGT-3′ Rv: 5′-CAAAAGCAATTGCCTCTTCAGC-3′; Human *MYH14*, Fw: 5′-GCCCTGGAAGCCGACCAT-3′ Rv: 5′-GAAGGCACCCACACGAGAC-3′; Human *ACTN1*, Fw: 5′-CACTTTGACCGGGATCACTCC-3′ Rv: 5′-TCATCCGTGTCCATCATGCC-3′; Human *ACTB*, Fw: 5′-GAGCACAGAGCCTCGCCTTT-3′ Rv: 5′-TCATCATCCATGGTGAGCTGG-3′; Human *GAPDH*, Fw: 5′-ACAGTCAGCCGCATCTTCTTT-3′ Rv: 5′-CAATACGACCAAATCCGTTGACT-3′ [10].

Quantification of mRNA expression of the target genes was calculated with the ΔΔCT method and mRNA expression for the genes of interest was normalised relative to GAPDH mRNA expression.

### mRFP-GFP-LC3 mouse ageing study

mRFP-GFP-LC3 mice and wild-type littermates were aged to 2, 12, 18 and 24 months. Following anesthetisation using standard procedures, mice were perfused with 10 ml PBS followed by 40 ml 4% PFA. Perfusion was performed at a rate of 4 ml/min. At this point the mouse tails were collected for genotyping. Following successful fixation, brains were removed and placed in 10 ml tubes with 4% PFA. After 3–4 h, the PFA was removed and 30% sucrose in deionized H_2_O was added. The brains in sucrose were placed in 4 °C until the brains sank to the bottom of the tube. The two hemispheres were separated and each hemisphere was placed on OCT embedding medium (ThermoScientific), frozen on dry ice and stored at −80 °C.The fixed mRFP-GFP-LC3 brains were sectioned sagittally into 8 μm thick sections on a cryostat (Leica CM3050S). Sections were collected on Superfrost slides (ThermoScientific) and either air-dried for 1 h for immunostaining or stored at −80 °C until further use. To reduce autofluorescence, the brains were subjected to Sudan Black B (SBB) staining. The brain sections were first air-dried for 1 h before being dipped into containers with 0.05% SBB in 70% methanol for 7 min. The sections were air-dried for 10 min before being mounted with the nuclear counterstain DAPI (1 μg/ml; Thermofisher) in antifadent mountant solution (Citifluor). The brain sections were imaged with a 63x objective with the LSM710 confocal microscope. For each mouse, five images were taken of the motor cortex. mRFP positive puncta that overlapped with GFP positive puncta (GFP/mRFP-double positive vesicles) were counted as autophagosomes, while puncta that were only positive for mRFP (mRFP-single positive vesicles) were counted as autolysosomes.

### Wild-type mouse ageing study

Wild-type mice were aged to 2, 12, 18 and 24 months of age. The mice were sacrificed and their brains dissected. Coronal sections of the left brain hemispheres were cut using a brain matrix (WPI) for precision. The brain area of interest was identified using a mouse brain atlas. The rills in the matrix enabled cutting a selected 2 mm section of each brain using a razorblade. The chosen area contains motor cortex, somatosensory cortex and striatum. The tissue was transferred to a glass homogeniser and homogenised with 500 μl Trizol (Ambion) in a fume hood on ice. The lysate was passed through a 27G syringe ten times and transferred to a 1.5 ml tube. The lysate was centrifuged at 13,200 rpm for 3 min at 4 °C and RNA was extracted using the RNA extraction kit (Qiagen, RNeasy Mini Kit) as per manufacturer’s protocol. Following elution of RNA, the concentration and purity were determined using a NanoDrop 2000c spectrophotometer. The spectrophotometer analysed the A260/280 and A260/230 ratios, which must be above 1.8 for quality RNA. The RNA was stored at −80 °C until 1 μg in 60 μl buffer was shipped deCODE Genetics (Iceland) for RNA sequencing (RNA-Seq).

deCODE Genetics assessed the quality and quantity of the total RNA using a LabChip GX instrument (Perkin Elmer) with the 96-well RNA kit. Indexed sequencing libraries were prepared using the TruSeq RNA sample preparation v2 kit (Illumina; 96-well plate format). In short, between 0.1–1 µg of total RNA was used for poly-A mRNA capture using oligo-dT attached magnetic beads. cDNA synthesis was done using SuperScript II and random hexamer priming. End-repair, 3′-adenylation, ligation of indexed adaptors and PCR amplification was performed as described by Illumina. Quantity and quality of each sequencing library were assessed using the LabChip GX, followed by standard dilutions and sample storage at −20 °C. Further quality assessment was performed by pool sequencing (≤24 samples/pool) on a MiSeq instrument in order to optimise cluster densities and assess insert size, sample diversity etc. Pooled samples (4 samples per pool) were clustered on paired-end (PE) flowcells using a cBot instrument (Illumina). Sequencing was performed on HiSeq 2500 using v4 SBS sequencing kits with readlengths at 2 × 125 cycles. Primary processing and base calling was performed using HCS and RTA (Illumina). Demultiplexing and generation of FASTQ files were performed using scripts from Illumina (bcl2fastq v.1.8).

### RNA sequencing data analysis

After receiving the mouse RNA-Seq data from deCODE Genetics, the reads were aligned to a mouse reference genome (UCSC assembly mm10). Data frames for rpkm (Reads Per Kilobase of transcript per Million) values for the genes of interest were generated in R [59]. Scatter plots with linear regression lines were then generated in R for a selection of genes (*Sorbs3*, *Camk4*, *Eda2r, Tuba1a, Yap1, Taz, Mlc2*, *Myh10*, *Birc2*, *Erbb4*, *Runx2*, *Ccnd1* and *Dab2*). Overall significance of the regression analysis was established by F-test performed in R, with the adjusted coefficient of determination (adjusted R2) also calculated.

RNA sequencing data from ‘neuropathological normal’ human frontal cerebral cortex and hippocampal tissue were obtained from the Genotype-Tissue Expression (GTEx) Consortium (Release 6) [39]. Donors labelled as follows in the subject metadata file were excluded: amyotrophic lateral sclerosis, Alzheimer’s disease OR dementia, Alzheimer’s disease, dementia with unknown cause, major depression (unipolar depression, major depressive disorder), active encephalitis, Creutzfeldt Jakob relatives, active meningitis, multiple sclerosis, Parkinson’s disease, Reyes syndrome, schizophrenia, syphilis infection, unexplained weakness. Donors were also excluded when cause of death was annotated with the following ICD10 codes: C70 to C72 (malignant neoplasms of brain and other parts of central nervous system), F00 to F99 (mental and behavioural disorders), G00 to G99 (diseases of the nervous system) and I60 to I69 (cerebrovascular disease). Rpkm values for the genes of interest in both tissues were provided by the GTEx Consortium (Release 6) [39]. Data frames for rpkm values and sample metadata were generated in R for both tissues, with the rpkm data filtered to exclude mitrochondrial and Y chromosome genes. Scatter plots with linear regression lines were then generated in R for a selection of genes (*SORBS3*, *CAMK4*, *EDA2R, TUBA1A, YAP1, TAZ, MLC2*, *MYH10*, *BIRC2*, *ERBB4*, *RUNX*2, *CCND1* and *DAB2*). Overall significance of the regression analysis was established by F-test performed in R, with the adjusted coefficient of determination (adjusted R2) also calculated.

### Statistical analysis

Aside from the RNA sequencing data analysis, initial data processing was performed in Excel (Microsoft), before statistical testing and graph construction using PRISM software (version 5.01, GraphPad). As appropriate, PRISM was used to perform: paired t-tests (equivalent to one-sample t-tests for normalised western blotting data; see below), Student’s t-tests, and one-way ANOVAs followed by Tukey’s multiple comparison test. Error bars indicate the standard deviation (SD) or standard error of the mean (SEM), as appropriate to the data presented (see figure legends). Unless otherwise stated, ns = *p* > 0.05 (*p* value), * = *p* < 0.05, ** = *p* < 0.01, *** = *p* < 0.001.

Western blotting data were typically generated from at least three independent experiments in biological triplicate. In order to normalise these data, protein levels (‘protein X’) in the experimental conditions were expressed relative to loading control protein levels and mean protein X/loading control in the experimental conditions normalised to mean protein X/loading control in the control condition. Accordingly, while there is within-condition variability (represented by the SD) between technical replicates, within-condition variability between control values generated from independent experiments is abolished (all mean values are 1). Within-condition variability (represented by the SEM) remains between protein X/loading control values in the experimental condition(s). Paired t-tests performed on these normalised data sets are therefore equivalent one sample t-tests. When testing perturbations in the presence or absence of Bafilomycin A1 (LC3-II blots), we have analysed the DMSO and Bafilomycin data separately (as one does not always get similar directions of changes with perturbants in both conditions)—thus, we have normalised the control values separately for DMSO and Bafilomycin to 1 in each experiment for statistical analysis. However, in the graphs, we have shown data where we have only normalised the control data in DMSO to 1, so that the effects of the Bafilomycin treatments can be clearly seen.

## Supplementary information

Supplementary Figures and legends

## Data Availability

The human RNA-Seq data analysed in this study is already publicly available via the Genotype-Tissue Expression (GTEx) Portal (see: https://www.gtexportal.org/home/) Following acceptance of this study for publication, the raw mouse RNA-Seq data will be deposited in an INSDC (DDBJ, ENA or GenBank) repository. The mouse RNA-Seq data for this study have been deposited in the European Nucleotide Archive (ENA) at EMBL-EBI under accession number PRJEB48346. All other data generated and/or analysed during this study are included in the main paper and supplementary information files.
