# Supplementary material for: Vinexin contributes to autophagic decline in brain ageing across species
Source: Cell Death Differ. 2021 Nov 30;29(5):1055–70. doi: 10.1038/s41418-021-00903-y (PMC9090768; doi:10.1038/s41418-021-00903-y)
Supplement: Supplementary file 1 — Supplementary Figures and legends [file 41418_2021_903_MOESM1_ESM.pdf]

Supplementary figure 1.

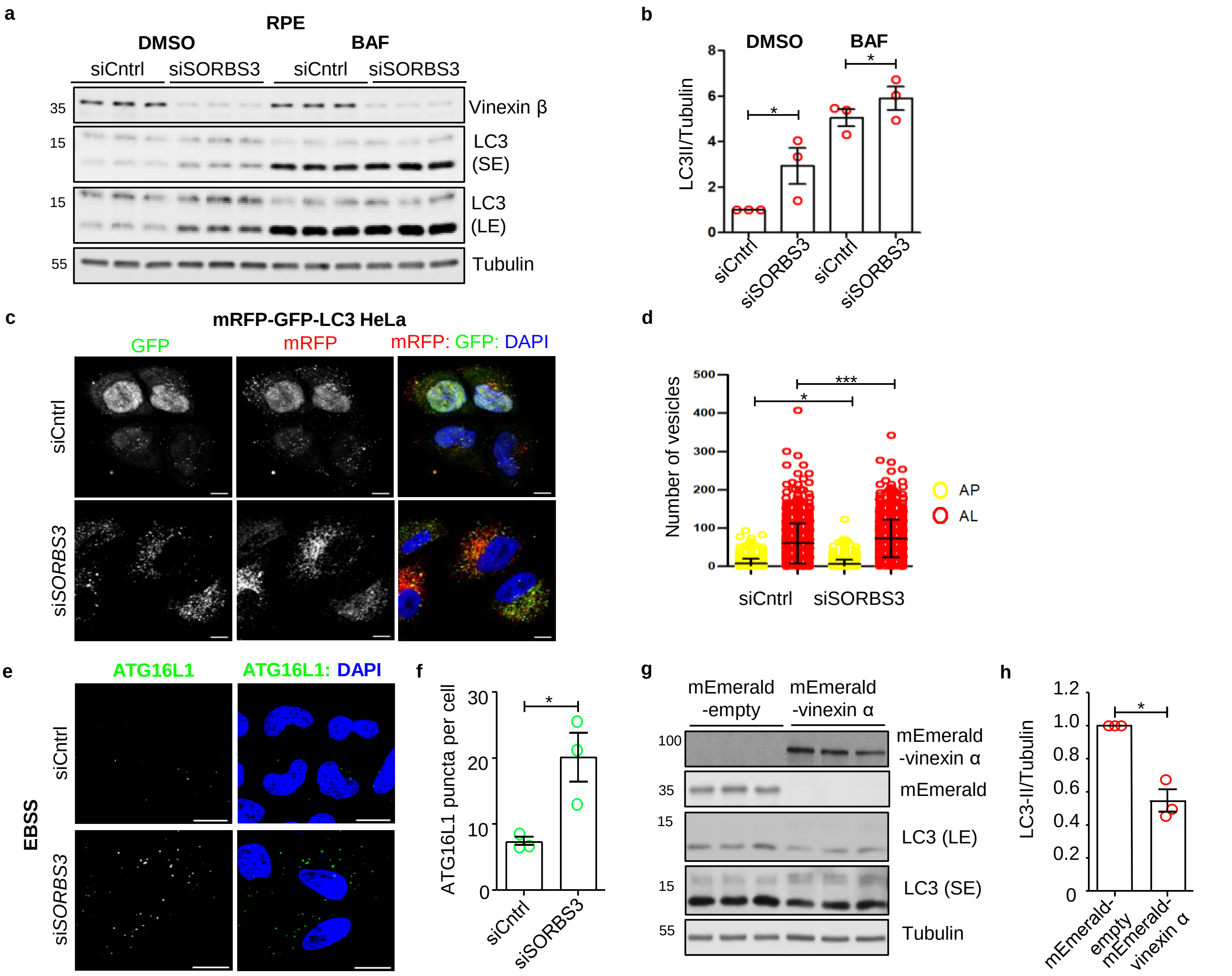

Supplementary figure 1. Vinexin negatively regulates autophagy.

a. RPE cells were depleted of vinexin beta using an individual siRNA oligonucleotide against *SORBS3* (si*SORBS3*). Cells were treated with bafilomycin A1 (BAF; 400 nM) or DMSO vehicle control for 4 hours. Endogenous tubulin, LC3 and vinexin beta protein levels were examined by western blotting. Representative blot from 3 independent experiments per cell line are shown. SE = short exposure; LE = long exposure; molecular weights shown in kDa.
b. Quantification of 3 independent experiments per cell line is shown. LC3-II (lower band of LC3 doublet) levels are expressed relative to tubulin loading control and normalised to LC3-II/tubulin in control siRNA (siCntrl) treated cells. * = $p < 0.05$ by 2-tailed paired t-test. Error bars indicate SEM
c. HeLa cells stably expressing GFP-mRFP-LC3 were depleted of vinexin beta, as in a. Representative confocal images from 3 independent experiments. Error bars indicate SEM.
d. Quantification of the representative experiment described in c. AP (autophagosomes) = GFP, AL(autolysosomes) = mRFP-single positive vesicles. n = 709 (siCntrl, DMSO), 828 (si*SORBS3*, DMSO). * = $p < 0.05$, *** = $p < 0.001$ by 2-tailed Student's t-test. Error bars indicate SD.
e. HeLa cells were depleted of vinexin beta as in a. Cells were starved in Earle's Balanced Salt Solution (EBSS) for 4 hours. Endogenous ATG16L1 was examined by confocal microscopy. Representative images from 3 independent experiments are shown. Green = ATG16L1; blue = DAPI. Scale bars indicate 20 µm.
f. ATG16L1 puncta were counted from confocal images acquired as described in e. using ImageJ software. Quantification of 3 independent experiments is shown. * = $p < 0.05$ by 1-tailed paired t-test. Error bars indicate SEM.
g. HeLa cells were transfected with mEmerald-vinexin alpha or empty vector control (mEmerald-empty) for 48 hours. Levels of the transfected proteins were examined by western blotting, as well as endogenous tubulin and LC3 protein levels. Representative blots from 3 independent experiments are shown. SE= short exposure; LE = long exposure; molecular weights shown in kDa
h. Quantification of 3 independent experiments described in g. LC3-II (lower band of LC3 doublet) levels are expressed relative to tubulin loading control and normalised to LC3-II/tubulin in mEmerald-empty transfected cells. * = $p < 0.05$ by 2-tailed paired t-test. Error bars indicate SEM.

Supplementary figure 2.

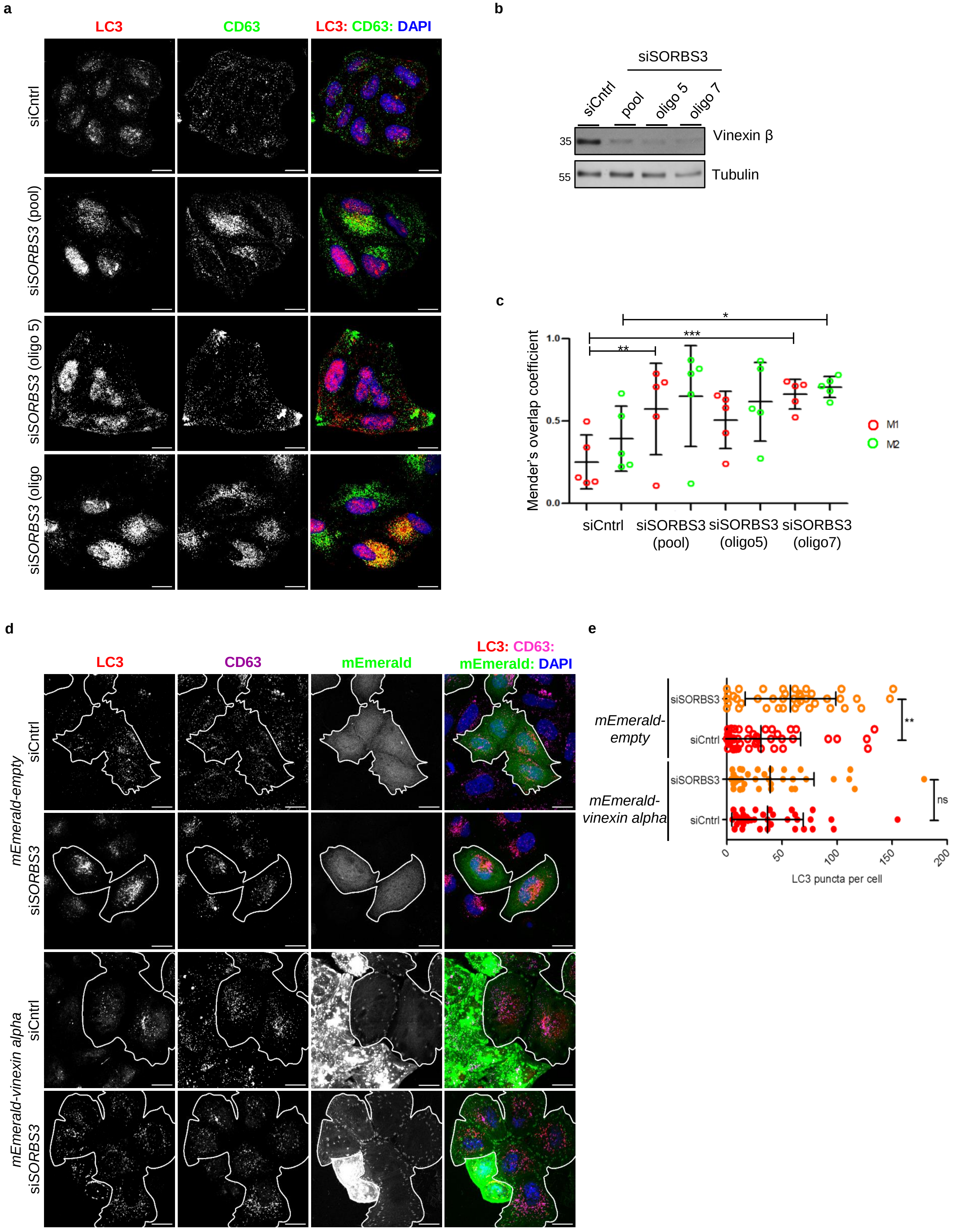

Supplementary figure 2. vinexin depletion using independent siRNA oligonucleotides upregulates autophagy, whereas vinexin overexpression ameliorates the increased autophagy caused by si*SORBS3* treatment.

a. HeLa cells were depleted of vinexin beta using a pool of 4 siRNA oligonucleotides against *SORBS3* [si*SORBS3* (pool)] and two individual siRNA oligonucleotides against SORBS3 [si*SORBS3* (oligo 5) and si*SORBS3* (oligo 7)]. Endogenous LC3 and CD63 were examined by immunofluorescence and confocal microscopy. Representative images from 2 independent experiments are shown. Red = LC3 (Alexa Fluor 568); green = CD63 (Alexa Fluor 488); blue = DAPI. Scale bars indicate 20 µm.
b. As part of the same experiments as a., Endogenous tubulin and vinexin beta protein levels were examined by western blotting. A representative blot from 2 independent experiments is shown. Molecular weights shown in kDa.
c. The proportion of CD63-positive pixels colocalising with LC3-positive pixels (Manders' colocalisation coefficient, M1) and proportion of LC3-positive pixels colocalising with CD63-positive pixels (Manders' colocalisation coefficient, M2) were determined from confocal images using Volocity software. Quantification of the representative experiment shown in a. * = $p < 0.05$, ** = $p < 0.01$, *** = $p < 0.001$ by one-way ANOVA followed by Tukey's multiple. comparison test. n = 47 (siCntrl), 27 [siSORBS3 (pool)], 25 [siSORBS3 (oligo 5)], 29 [siSORBS3 (oligo 7)]. Error bars indicate SD.
d. HeLa cells were depleted of vinexin beta using an individual siRNA oligonucleotide against SORBS3 (si*SORBS3*; oligo 7). siCntrl and si*SORBS3* treated cells were transfected with mEmerald-vinexin alpha or empty vector control (mEmerald-empty) for 48 hours. Endogenous LC3 and CD63, as well as mEmerald, were examined by immunofluorescence and confocal microscopy. Representative images from 3 independent experiments are shown. Red = LC3 (Alexa Fluor 568); purple = CD63 (Alexa Fluor 647); green = mEmerald; blue = DAPI. Scale bars indicate 20 µm.
e. LC3 puncta were counted from confocal images acquired as described in a. using ImageJ software. Quantification of the representative experiment shown in a. ns = $p > 0.05$; ** = $p < 0.01$ by one-way ANOVA followed by Tukey's multiple comparison test. n = 45 (mEmerald-empty, siCntrl), 40 (mEmerald-empty, si*SORBS3*), 41 (mEmerald-empty, si*SORBS3*), 38 (mEmerald-vinexin alpha, si*SORBS3*). Error bars indicate SD.

Supplementary figure 3.

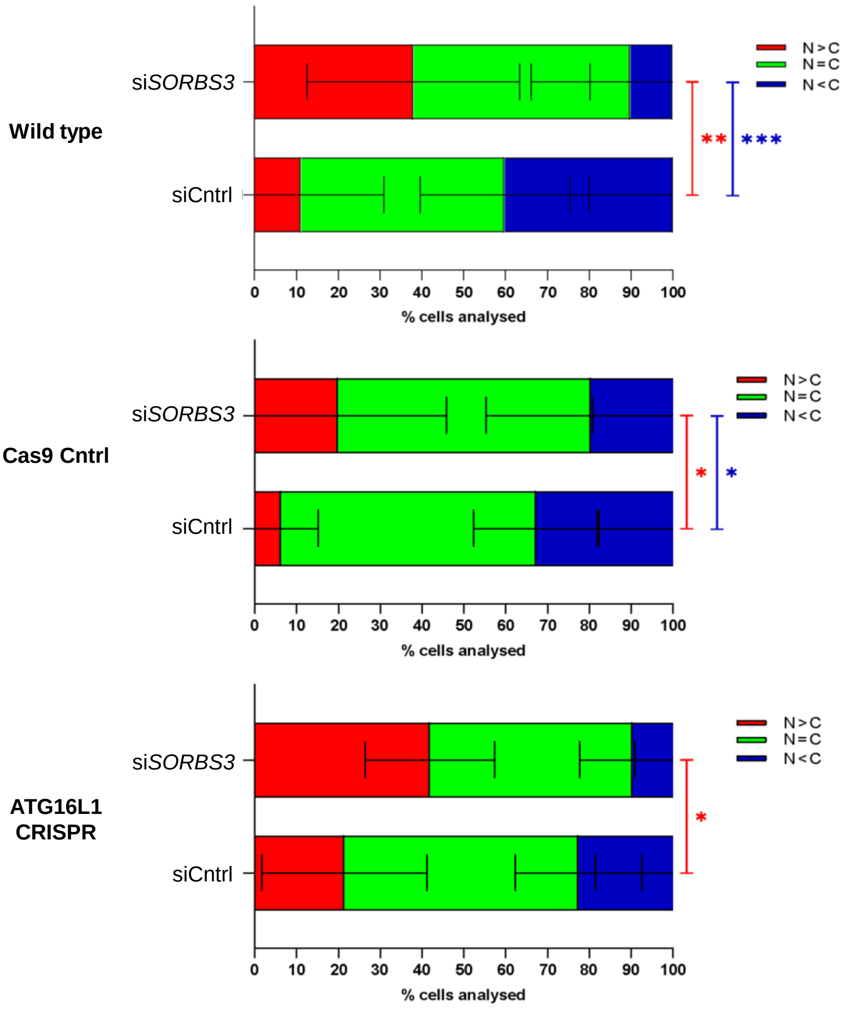

Supplementary figure 3. Vinexin depletion increases autophagy through YAP/TAZ nuclear localisation upstream to upregulating autophagy.

Wild type, Cas9 control (Cntrl) and ATG16L1 CRISPR HeLa cells were depleted of vinexin beta using an individual siRNA oligonucleotide against SORBS3 (*siSORBS3*). Endogenous YAP/TAZ were examined by immunofluorescence and confocal microscopy. Images from 3 independent experiments per cell line are shown in Fig. 2a. This figure shows quantified of images from these representative experiments. Cells with predominantly nuclear YAP/TAZ (N > C), YAP/TAZ equally distributed between nucleus and cytosol (N = C) and predominantly cytosolic YAP/TAZ (C > N) where manually quantified. * = $p < 0.05$; ** = $p < 0.01$; *** = $p < 0.001$ by 2-tailed Student's t-test. Red asterisks represent p value for N > C; blue asterisks represent N < C p vale. n = 242 (wild type, siCntrl); 203 (wild type, siSORBS3); 313 (Cas9 Cntrl, siCntrl); 216 (Cas9 Cntrl, siSORBS3), 132 (ATG16L1 CRISPR, siCntrl); 115 (ATG16L1 CRISPR, siSORBS3). Error bars indicate SD.

Supplementary figure 4.

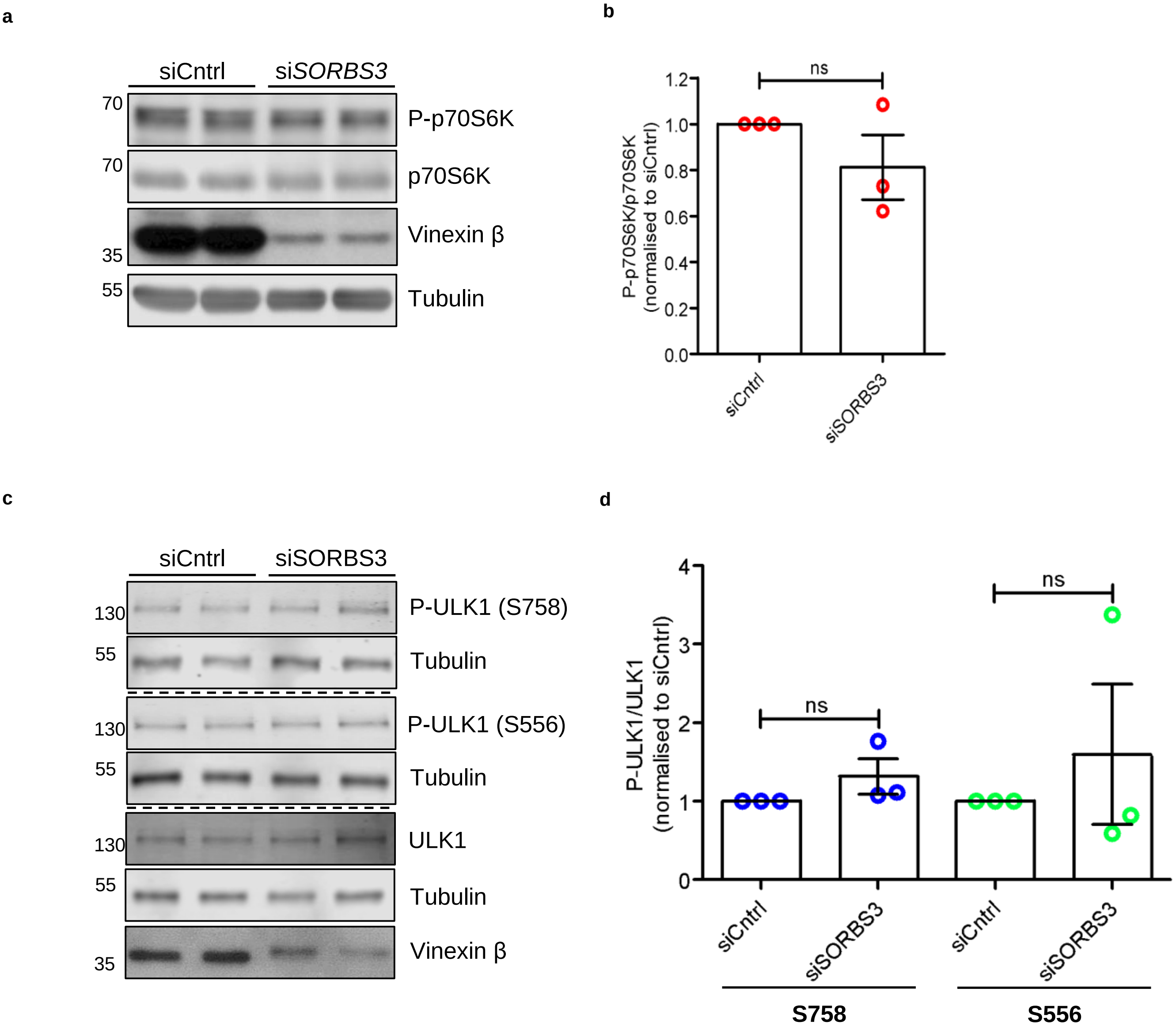

Supplementary figure 4. Vinexin depletion does not impact canonical autophagy signalling pathways (mTOR and ULK1).

a. HeLa cells were depleted of vinexin beta using a pool of 4 siRNA oligonucleotides against SORBS3 (siSORBS3). Endogenous total ribosomal protein S6 kinase (p70S6K), p70S6K phosphorylated at threonine 389 (P-p70S6K), tubulin and vinexin beta protein levels were examined by western blotting. Representative blots from 3 independent experiments are shown. Molecular weights in kDa.
b. Quantification of 3 independent experiments. P-p70S6K and p70S6K are expressed relative to tubulin loading control, the ratio of P-p70S6K/tubulin: p70S6K/tubulin taken and then normalised to P-p70S6K/p70S6K in siCntrl treated cells. ns = $p > 0.05$ by 2-tailed paired t-test. Error bars indicate SEM.
c. HeLa cells were depleted of vinexin beta using an individual siRNA oligonucleotide against SORBS3 (siSORBS3). Endogenous total ULK1, ULK1 phosphorylated at serine 758 [P-ULK1 (S758)], ULK1 phosphorylated at serine 556 [P-ULK1 (S556)], tubulin and vinexin beta protein levels were examined by western blotting. Representative blot from 3 independent experiments are shown. Molecular weights in kDa.
d. Quantification of 3 independent experiments. P-ULK1 and ULK1 are expressed relative to tubulin loading control, the ratio of P-ULK1/tubulin: ULK1/tubulin taken and then normalised to P-ULK1/ULK1 in siCntrl treated cells. ns = $p > 0.05$ by 2-tailed paired t-test. Error bars indicate SEM.

Supplementary figure 5.

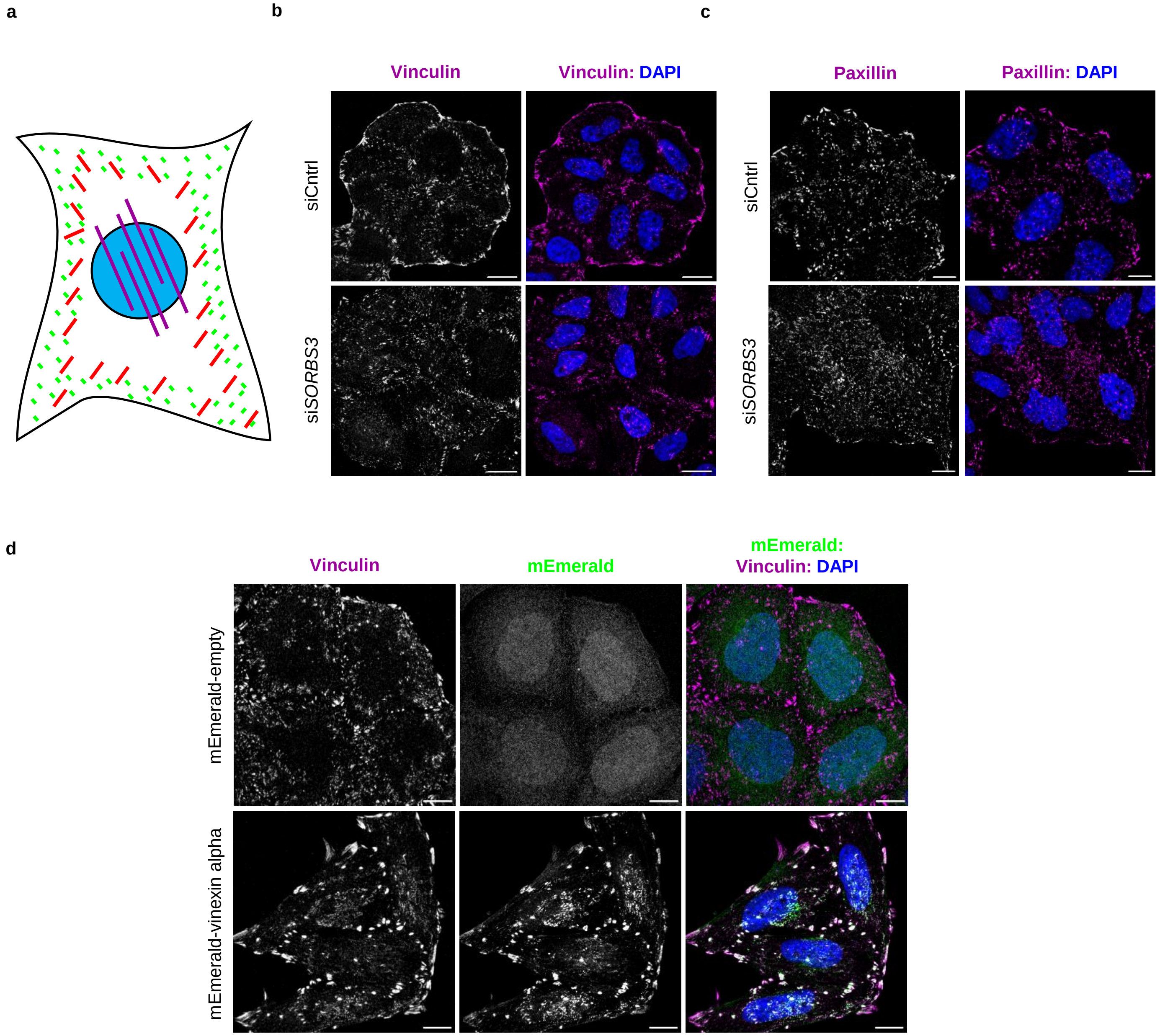

Supplementary figure 5. Focal adhesion destabilisation and stabilisation correlate with vinexin beta depletion and vinexin alpha overexpression, respectively.

a. Schematic diagram depicting the three recognised focal adhesion types: 1. small focal complexes (green), 2. larger focal adhesions (red) and 3. large, centrally-located fibrillar adhesions (purple).
b. HeLa cells were depleted of vinexin beta using an individual siRNA oligonucleotide against *SORBS3* (si*SORBS3*). Endogenous vinculin (a canonical focal adhesion protein) was examined by confocal microscopy. siSORBS3 destabilised larger focal adhesions and favoured small focal complexes. Representative images from 3 independent experiments are shown. Purple (Alexa Fluor 647) = vinculin; blue = DAPI. Scale bars indicate 20 µm.
c. HeLa cells were depleted of vinexin beta, as in b. Endogenous paxillin (another canonical focal adhesion protein) was examined by confocal microscopy. Again, siSORBS3 destabilised larger focal adhesions and favour small focal complexes. Representative images from 3 independent experiments are shown. Purple (Alexa Fluor 647) = paxillin; blue = DAPI. Scale bars indicate 10 µm.
d. HeLa cells were transfected with mEmerald-vinexin alpha or empty vector control (mEmerald-empty) for 48 hours. Endogenous vinculin, as well as mEmerald, were examined by immunofluorescence and confocal microscopy. Vinexin alpha overexpression stabilised larger focal adhesions. Representative images from 3 independent experiments are shown. Purple = vinculin (Alexa Fluor 647); green = mEmerald; blue = DAPI. Scale bars indicate 10 µm.

Supplementary figure 6.

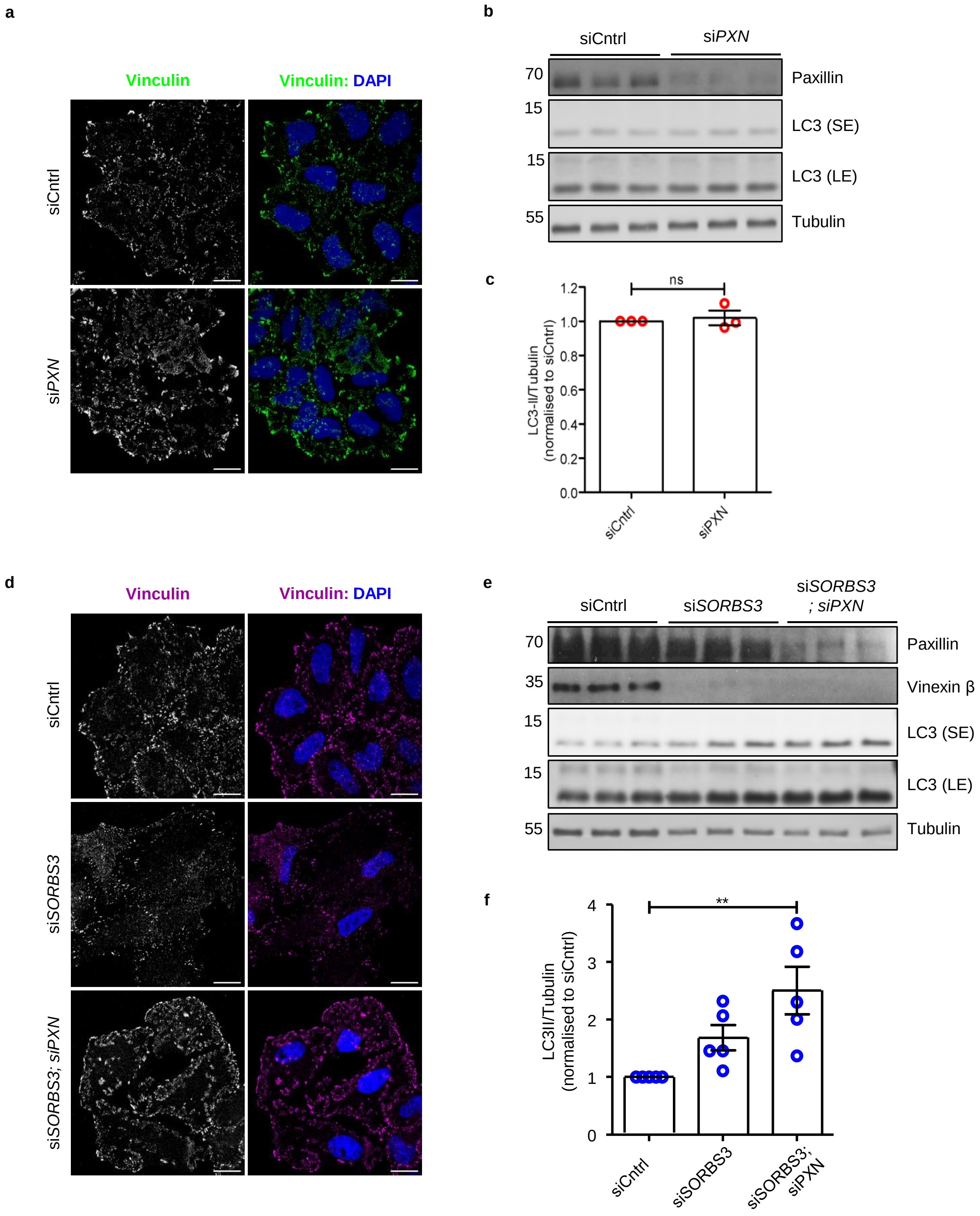

Supplementary figure 6. Focal adhesion destabilisation and stabilisation are not causally related to autophagy upregulation and downregulation in HeLa cells.

a. HeLa cells were depleted of paxillin using a pool of 4 siRNA oligonucleotides against *PXN* (si*PXN*). Endogenous vinculin was examined by confocal microscopy. Paxillin depletion stabilised larger focal adhesions, much like vinexin alpha overexpression (Sup. Fig. 5d). Representative images from 3 independent experiments are shown. Green (Alexa Fluor 488) = vinculin; blue = DAPI. Scale bars indicate 20 µm.
b. HeLa cells were depleted of paxillin, as in a. Endogenous tubulin, LC3 and paxillin protein levels were examined by western blotting. Despite stabilising larger focal adhesions (much like vinexin alpha overexpression), unlike vinexin alpha overexpression, paxillin depletion failed to downregulate autophagy. Representative blots from 3 independent experiments are shown. SE = short exposure; LE = long exposure; molecular weights shown in kDa.
c. Quantification of 3 independent experiments. LC3-II (lower band of LC3 doublet) levels are expressed relative to tubulin loading control and normalised to LC3-II/tubulin in control siRNA (siCntrl) treated cells. ns = $p > 0.05$ by 2-tailed paired t-test. Error bars indicate SEM.
d. HeLa cells were depleted of vinexin beta using an individual siRNA oligonucleotide against *SORBS3* (si*SORBS3*; oligo 7) and paxillin using a pool of 4 siRNA oligonucleotides against *PXN* (si*PXN*). Endogenous vinculin was examined by confocal microscopy. Paxillin depletion ameliorated the focal adhesion destabilisation caused by vinexin beta depletion. Representative images from 3 independent experiments are shown. Purple (Alexa Fluor 647) = vinculin; blue = DAPI. Scale bars indicate 20 µm.
e. HeLa cells were depleted of vinexin beta and paxillin, as in d. Endogenous tubulin, LC3, vinexin beta and paxillin protein levels were examined by western blotting. Despite ameliorating the focal adhesion destabilisation caused by vinexin beta depletion, paxillin depletion did not ameliorate the increase in LC3-II caused by vinexin beta depletion. Representative blots from 5 independent experiments are shown. SE = short exposure; LE = long exposure; molecular weights shown in kDa.
f. Quantification of 5 independent experiments. LC3-II (lower band of LC3 doublet) levels are expressed relative to tubulin loading control and normalised to LC3-II/tubulin in siCntrl treated cells. ** = $p < 0.01$ by one-way ANOVA followed by Tukey's multiple comparison test. Error bars indicate SEM.

Supplementary figure 7

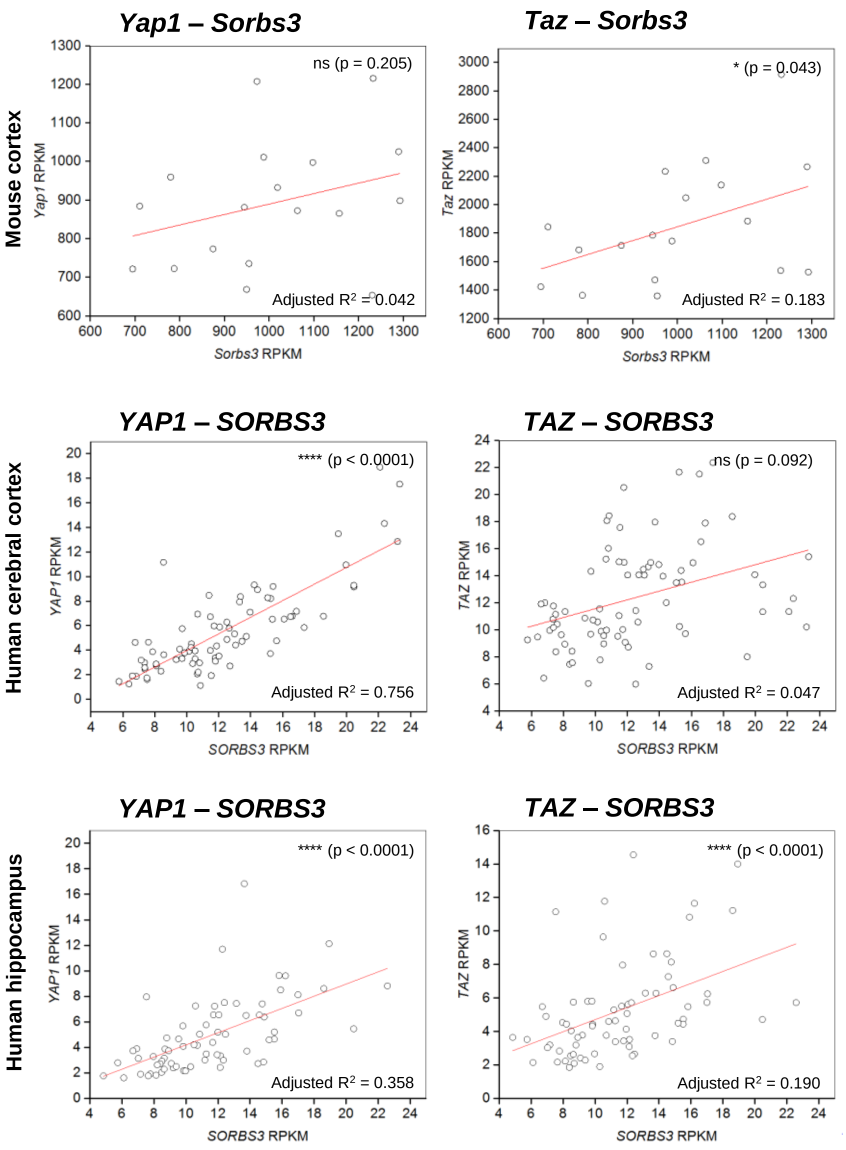

Supplementary figure 7. Correlation of YAP/TAZ and SORBS3 mRNA expression in mouse and human brain tissues.

(top) Correlation plot of *Yap1/Taz* and *Sorbs3* mRNA expression determined by RNA sequencing of samples from wild-type mouse brain tissue (combined motor cerebral cortex, somatosensory cerebral cortex and striatum). The adjusted coefficient of determination (adjusted R^2) and the corresponding p-value for the overall significance of the regression (F-test) are displayed on the scatter plot (n = 18, RPKM = Reads Per Kilobase of transcript per Million).

(bottom) Correlation plot of *YAP1/TAZ* and *SORBS3* mRNA expression determined by RNA sequencing of samples from 'neuropathological normal' human frontal cerebral cortex and hippocampus plotted against chronological age of the donors in years. The adjusted coefficient of determination (adjusted R^2) and the corresponding p-value of the overall significance of the regression analysis (F-test) are displayed on the scatter plot (n = 77 and 70, respectively; RPKM = Reads Per Kilobase of transcript per Million).

Supplementary figure 8

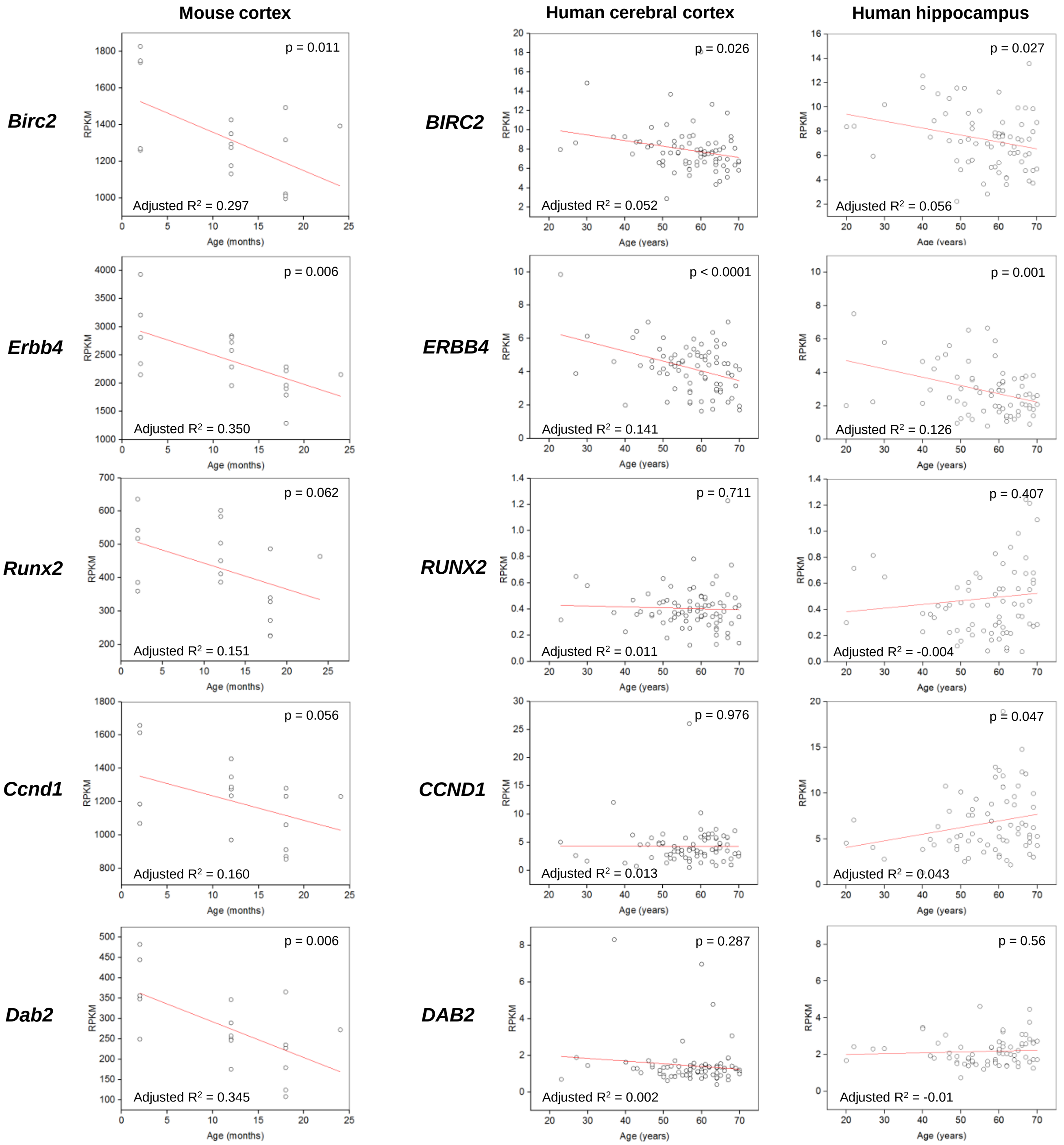

Supplementary figure 8. YAP/TAZ-TEAD target genes expression declines with age in mouse and human brain tissues.

(left) mRNA expression of *Birc2, Erbb4, Runx2, Ccnd1* and *Dab2* determined by RNA sequencing of samples from wild-type mouse brain tissue (combined motor cerebral cortex, somatosensory cerebral cortex and striatum) plotted against chronological age of animals in months. The adjusted coefficient of determination (adjusted R2) is displayed to two significant figures on the scatter plot (bottom left). Overall significance of the regression analysis was established by F-test and the p value displayed on the scatter plot (top right). n = 18. RPKM = Reads Per Kilobase of transcript per Million.

(right) mRNA expression of *BIRC2, ERBB4, RUNX2, CCND1* and *DAB2* determined by RNA sequencing of samples from ‘neuropathological normal’ human frontal cerebral cortex and hippocampus plotted against chronological age of the donors in years. The adjusted coefficient of determination (adjusted R2) is displayed to two significant figures on the scatter plot (bottom left). Overall significance of the regression analysis was established by F-test and the p value displayed on the scatter plot (top right). n = 77 (cerebral cortex) and 70 (hippocampus). RPKM = Reads Per Kilobase of transcript per Million.
